# Role of PDE4 Family in Cardiomyocyte Physiology and Heart Failure

**DOI:** 10.3390/cells14060460

**Published:** 2025-03-20

**Authors:** Ivan Sherstnev, Aleksandra Judina, Giovanni Battista Luciani, Alessandra Ghigo, Emilio Hirsch, Julia Gorelik

**Affiliations:** 1 Cardiac Section, National Heart and Lung Institute (NHLI), Faculty of Medicine, Imperial College London, Hammersmith Campus, Du Cane Road, London W12 0NN, UK; ivan.sherstnev_02@univr.it (I.S.); a.judina18@imperial.ac.uk (A.J.); 2 Department of Surgery, Dentistry, Pediatrics and Gynecology, Division of Cardiac Surgery, University of Verona, 37126 Verona, Italy; giovanni.luciani@univr.it; 3 Department of Molecular Biotechnology and Health Sciences, Molecular Biotechnology Center “Guido Tarone”, University of Torino, 10126 Torino, Italy; alessandra.ghigo@unito.it (A.G.); emilio.hirsch@unito.it (E.H.)

**Keywords:** Phosphodiesterase 4, cAMP signalling, cardiomyocyte, β-adrenergic signalling, arrhythmia, cAMP compartmentation, A-kinase-anchoring protein, Phosphodiesterase inhibitor

## Abstract

Phosphodiesterase 4 (PDE4) is a key regulator of cyclic adenosine monophosphate (cAMP) signalling in cardiomyocytes, controlling contractility, calcium handling, and hypertrophic responses. PDE4 provides spatial and temporal precision to cAMP signalling, particularly under β-adrenergic stimulation, through its compartmentalised activity in subcellular nanodomains, including the sarcoplasmic reticulum, plasma membrane and nuclear envelope. This review highlights the cardiac PDE4 isoforms PDE4A, PDE4B and PDE4D, focusing on their distinct localisation and contributions to cardiac physiology and pathophysiology, particularly in heart failure and arrhythmias. Although PDE4 plays a smaller role in overall cAMP hydrolysis in human hearts than in rodents, its compartmentalised function remains critical. Recent therapeutic advances have shifted from pan-PDE4 inhibitors to isoform-specific approaches to enhance efficacy while minimising systemic toxicity. We discuss the potential of selective PDE4 modulators, gene therapies and combination strategies in restoring cAMP compartmentation and preventing maladaptive cardiac remodelling. By integrating rodent and human studies, this review underscores the translational challenges and therapeutic opportunities surrounding PDE4, positioning it as both a key regulator of cardiac signalling and a promising target for heart failure therapies.

## 1. Introduction

### 1.1. PDE Superfamily

Mammalian cells express 21 PDE genes, grouped into 11 superfamilies, collectively generating over 100 isoforms [1,2]. These isoforms exhibit tissue-specific and subcellular distribution, including within cardiomyocytes, allowing them to regulate specific GPCR signalling pathways and their associated cellular functions [3,4,5].

cAMP is a key second messenger in cardiomyocytes, generated by adenylate cyclases following β-adrenergic receptor activation. cAMP regulates contractility, heart rate and relaxation via protein kinase A (PKA) and other effector proteins [6,7]. PKA is a heterotetramer consisting of two regulatory (PKA-R) and two catalytic (PKA-C) subunits [8]. The regulatory subunit exists in two major isoforms, PKA-RI and PKA-RII, which differ in their biochemical properties, subcellular localisation and regulatory roles [9,10]. PKA-RI is predominantly cytosolic and more sensitive to cAMP, while PKA-RII is anchored to specific subcellular sites via AKAPs, allowing localised, compartmentalised signalling [9,11,12].

cGMP, synthesised by guanylate cyclases in response to nitric oxide or natriuretic peptides, regulates vasodilation, myocardial relaxation and antihypertrophic signalling via protein kinase G. Its interplay with cAMP, mediated by PDEs, modulates cAMP hydrolysis, ensuring precise regulation of cardiac signalling [6,13].

Based on substrate specificity, PDEs are classified into three main categories, as presented in Table 1.

In the heart, eight PDE families have been identified: PDE1 [41], PDE2 [32], PDE3 [35], PDE4 [42], PDE5 [43], PDE8 [19], PDE9 [44] and PDE10 [45] (Table 2). Nevertheless, the heart expresses mRNA for all PDEs except PDE6, though the importance of this is yet to be discovered [46].

### 1.2. PDE4 Structure and Function

PDE4 has garnered significant attention over the past few decades due to its prominent involvement in cardiac physiology and pathology. PDE4 uniquely hydrolyses cAMP with high specificity, positioning it as a central regulator of cAMP compartmentation within cardiomyocytes [67]. This precise regulation is crucial for maintaining cardiac function under both physiological and pathological conditions. The distinct roles of PDE4 isoforms in modulating β-adrenergic signalling [68,69,70], calcium handling [71,72] and hypertrophic responses [73,74] have highlighted their potential as therapeutic targets. Consequently, understanding the structural and functional nuances of PDE4 isoforms is pivotal for revealing new strategies to counteract cardiomyocyte maladaptive remodelling and develop innovative heart failure therapies.

**Figure 1:**
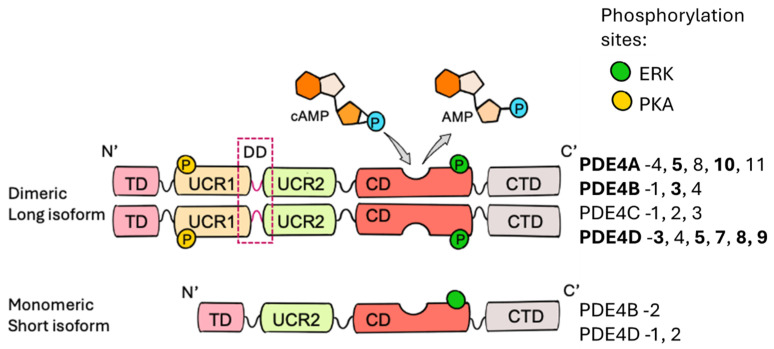
Structural organisation, classification, regulation and enzymatic activity of PDE4 long and short isoforms in the heart and cardiomyocytes. The diagram illustrates the domain architecture of dimeric long and monomeric short isoforms of PDE4. Long isoforms contain UCR1 and UCR2 domains, facilitating dimerisation via the dimerisation domain (DD, dashed box), a surrogate substrate of PKA phosphorylation, while short isoforms lack UCR1 and remain monomeric. The catalytic domain (CD, red) hydrolyses cAMP into AMP. Phosphorylation by ERK (green circle) and PKA (yellow circle) regulates PDE4 activity, affecting enzyme stability and function. The C-terminal domain (CTD) contributes to isoform-specific interactions within cells. Isoforms confirmed in cardiomyocytes are shown in bold.

The mammalian genome encodes four PDE4 genes—*PDE4A*, *PDE4B*, *PDE4C*, and *PDE4D* [14,15]—which, through alternative translational sites and splicing, yields approximately 25 distinct isoforms. Figure 1 illustrates the structural organisation, classification, regulation and enzymatic activity of PDE4 long and short isoforms in the heart and cardiomyocytes. Each isoform is potentially associated with specialised functions linked to specific subcellular localisations [3].

Based on the presence or absence of upstream conserved region (UCR) domains, the isoforms are classified as long, short or supershort forms [5]. The UCR domains, UCR1 and UCR2, are separated by the Linker Region 1 (LR1) and are crucial for the dimerisation of the enzyme [75,76]. Isoforms lacking UCR1—namely, the short and supershort forms—predominantly exist as monomers. The activity of PDE4 is regulated via the phosphorylation of Ser190 (for PDE4D) and Ser133 (for PDE4B) by PKA at the UCR1 domain [75,77,78,79,80,81], which enables PDE4 dimerisation, enhancing long isoform hydrolytic activity. The mitogen-activated protein kinase 1 (MAPK1, also known as ERK2) mediates phosphorylation at the C-terminal region, which causes an inhibitory effect on the long and supershort isoforms and activation of short isoforms [82,83,84,85,86]. This structural complexity underpins PDE4’s diverse roles in cardiac tissues, as detailed in the following sections.

**Figure 2:**
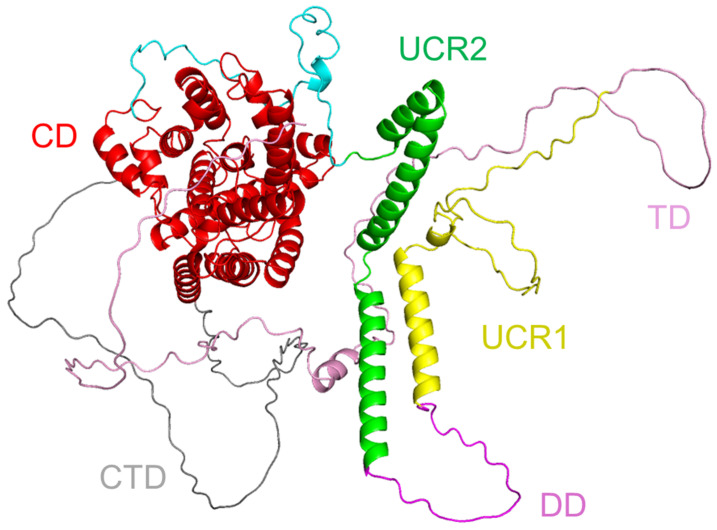
Three-dimensional structural model of human PDE4B protein. The structural organisation highlights key functional regions: catalytic domain (CD, red), C-terminal domain (CTD, grey), dimerisation domain (DD, magenta), targeting domain (TD, pink), upstream conserved region 1 (UCR1, yellow) and upstream conserved region 2 (UCR2, green). UniProt: Q07343; generated using AlphaFold 3 [88,89] protein structure database: AF-Q07343-F1-v4); domains were marked with PyMol 3.1.1 [90].

Structural insights into the regulation of PDE4 activity, especially PDE4B, highlight the role of its long isoforms, which contain the upstream conserved regions (UCR1 and UCR2). These domains mediate dimerisation and allosteric regulation, distinguishing long PDE4 variants from short and supershort forms. Using X-ray crystallography and biochemical approaches, the authors reveal that UCR2 of one subunit interacts with the catalytic domain of the opposite subunit, thereby modulating enzymatic activity. This structural arrangement explains the differential regulatory properties among PDE4 isoforms and offers a mechanistic understanding of PDE4 inhibition [75,87]. A 3D structural model (Figure 2) of human PDE4B was created to visualise this organisation based on domain annotations adapted from [75]. This model corresponds to the long isoform of PDE4B, as shown in Figure 1, which includes UCR1 and UCR2 domains involved in dimerisation and regulation.

## 2. PDE4 Expression and Function in the Heart: From Species Variability to Subcellular Dynamics

### 2.1. PDE4 Expression and Function in Cardiac Tissue Across Different Species

While PDE4 localisation and functionality are conserved across rodent and human cardiac tissues [47,71,91,92,93], significant differences exist in their contributions to overall cAMP-PDE activity. In the human heart, PDE4 accounts for approximately 10% of total cAMP-phosphodiesterase activity, markedly lower than the 40–60% observed in rat and mouse hearts [47,72,93,94]. Despite these quantitative differences, PDE4 is similarly localised to the Z-band in cardiomyocytes in both rodents and humans [47]. Moreover, in humans, PDE4 is tethered to macromolecular complexes that also involve the same enzymes as in rodent hearts, including the PLN/SERCA2 complex and β_1_AR complexes [47].

Functionally, PDE4 regulates β-AR stimulated LTCC activity, a role conserved across species [71,91,92]. It also modulates RyR2 phosphorylation mediated by PKA, contributing to the fine-tuning of calcium cycling and excitation–contraction coupling [93]. This conservation underscores the critical role of PDE4 in localised cAMP signalling in cardiomyocytes, while species-specific differences in PDE4’s contribution to total cAMP-PDE activity highlight the importance of careful translation of findings from rodent models to human cardiac physiology.

Thus, the differences in PDE4 activity are primarily explained by the increased activity of other PDEs in humans, in particular, PDE3A [63,95,96]. The distinct kinetic properties of these enzymes further explain their differential regulation. It was revealed that PDE3 has a Km value roughly 10 times lower than PDE4 in the ventricular tissue of humans and guinea pigs, indicating that PDE3 is more efficient at hydrolysing cAMP at lower concentrations, while PDE4 becomes more active when cAMP levels rise, such as during β-adrenergic stimulation [97,98]. Similarly, PDE8 exhibits an even lower Km than PDE3 and is also expressed in cardiac tissues [53,54,99,100,101], suggesting a complementary role in maintaining basal cAMP levels. This suggests that PDE3 and PDE8 primarily regulate cAMP levels under basal conditions, while PDE4 plays a more prominent role when cAMP concentrations rise, such as during β-adrenergic stimulation. This kinetic disparity implies that PDE4’s regulatory role becomes more significant at elevated intracellular cAMP concentrations, matching its higher Km value, while PDE3 maintains functional activity at lower cAMP levels. It is also interesting that when PDE3 is inhibited, PDE4 becomes more active and reduces the impact of catecholamines on cAMP and L-type Calcium currents (ICa,L) [71]. The variations in enzyme properties help explain differences in how specific PDE4 isoforms are expressed and how they function in different species, as described below.

In murine cardiac tissues, PDE4A, PDE4B and PDE4D are expressed in similar proportions, while PDE4C is not detected [48]. Across species, including mice, rats and humans, specific PDE4 variants show distinct patterns of expression and localisation. All PDE4 isoforms were detected in mouse, rat and human hearts by Western blotting using pan-PDE4 antibodies. For example, the predominant isoform of PDE4A is PDE4A10 (105–110 kDa), with smaller amounts of PDE4A5 also present. In the PDE4B subfamily, PDE4B3 (~95 kDa) is the most abundantly expressed variant. The PDE4D subfamily exhibits the greatest diversity, with isoforms such as PDE4D3, PDE4D8 and PDE4D9 forming primary protein bands at 91–95 kDa, while PDE4D5 and PDE4D7 are detected as minor bands at higher molecular weights [47,102,103]. Thus, in cardiomyocytes, PDE4A5 [47], PDE4A10 [47,104], PDE4B3 [47,105], PDE4D3 [47,106], PDE4D5 [47,106,107,108], PDE4D7 [47], PDE4D8 [47] and PDE4D9 [47] were confirmed. Notably, all cardiac PDE4 splicing variants identified so far belong to long isoforms [109]. This observation highlights the critical role of PKA and β-adrenergic receptor signalling in regulating their function, as long isoforms are known to interact with specific macromolecular complexes to modulate cAMP levels effectively.

Cardiac PDE4 isoforms modulate cAMP levels across multiple subcellular compartments in cardiomyocytes [7]. The non-specific inhibition of PDE4 leads to a significant increase in cAMP during βAR activation in mice [7,48,110], rats [91,111] and humans [72]. In human atrial myocytes, PDE4, particularly the PDE4D subtype, plays a crucial role during β-adrenergic stimulation, where its inhibition significantly impacts calcium handling and contractile response, potentially contributing to arrhythmogenic events [72]. This modulation of cAMP and calcium handling highlights the broader role of PDE4 in heart function, as will be discussed next.

Key insights were provided about the species-specific effects of PDE4 inhibition with rolipram. In rat cardiomyocytes, PDE4 inhibition reduced cAMP-PDE activity, whereas its effects in guinea pig and human cardiomyocytes were negligible. Notably, in rats, rolipram had no effect under basal conditions but significantly enhanced contractility following β-adrenergic stimulation with isoproterenol or forskolin, increasing cardiomyocyte shortening by approximately 25% and 30%, respectively. These findings suggest that PDE4 inhibition primarily impacts cAMP signalling during β-adrenergic activation, which may be more pronounced under stress or disease states [94]. These findings were extended by investigating rolipram in failing human ventricular myocardium, where PDE4 inhibition failed to enhance inotropic or lusitropic responses, even with β-blocker treatment. While PDE4 inhibition appears less impactful in the healthy human heart, its role may still be relevant in pathological conditions where cAMP signalling is dysregulated. These results highlight a critical species difference, underscoring the need for caution when extrapolating findings from rodent models to human physiology. However, it is important to note that differences may also influence the discrepancies between these studies in experimental models (e.g., isolated cardiomyocytes vs. whole myocardial tissue), conditions (e.g., β-adrenergic activation) and disease states. While PDE4 inhibition appears less impactful under baseline conditions in the human heart, its role may still be relevant in pathological states or during heightened cAMP signalling [112].

### 2.2. Regional Differences in PDE4 Activity

**Figure 3:**
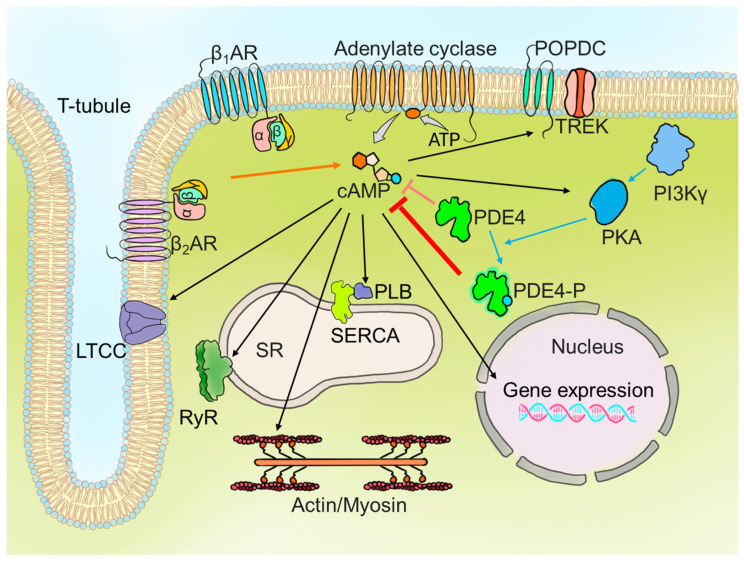
βAR regulation of cAMP signalling and PDE4 activity in cardiomyocytes. This schematic represents the βAR signalling cascade, which regulates cAMP levels and downstream targets in cardiomyocytes. Stimulation of β₁ARs and β₂ARs activates adenylate cyclase, converting ATP into cAMP. cAMP activates PKA, which phosphorylates various targets, including PLB and RyRs for calcium regulation, and LTCC to increase calcium influx. PDE4, regulated by AKAPs (particularly PI3Kγ), hydrolyses cAMP to AMP, modulating cAMP compartmentation and preventing excessive PKA activation. Phosphorylation of PDE4 further enhances its activity. PDE4 plays an important role in regulating nuclear gene expression and calcium handling, thereby regulating the contraction of cardiomyocytes.

βARs play a central role in regulating cAMP signalling, with PDE4 acting as a key modulator to ensure precise compartmentalisation and control of downstream cardiac responses [105,113,114]. Figure 3 illustrates the intricate network of βAR-cAMP-PDE4 signalling, highlighting PDE4’s role in shaping distinct cAMP pools that regulate various cardiac functions, including calcium handling, contractility and gene expression. Regional variations in PDE4 activity within the heart have significant implications for cardiac functions. Professor Gorelik’s research group reported that intra-chamber variability in PDE4 activity within the left ventricle limits β_2_AR-associated cAMP diffusion in basal but not apical cells. Reduced PDE4 enables higher cAMP diffusion to the PKA-RII domain, leading to enhanced contractile response [114]. These intra-chamber differences also extend to variations between the left and right ventricles, as discussed below.

Enhanced contractile response of right ventricular cardiomyocytes to catecholamine stimulation is attributed to elevated PDE3 and PDE4 activities in left ventricular cardiomyocytes [115]. This phenomenon may represent an adaptive mechanism to meet the increased oxygen demand during heightened physical activity by enhancing pulmonary circulation [116]. Specifically, canine models have shown a greater right ventricular cardiomyocyte response to βAR stimulation compared to left ventricular cells, associated with higher PDE3 and PDE4 activities in the left ventricle [115]. Beyond chamber-specific differences, PDE4 activity also varies within specific regions of the ventricular wall.

Cardiac chambers display distinct structural and functional characteristics, which may influence PDE4 activity. For example, a significant difference was demonstrated in calcium signalling between atrial and ventricular cardiomyocytes, linked to structural variations like the presence of T-tubules in ventricular cells [117]. The remodelling of right ventricular cardiomyocytes under conditions such as pulmonary hypertension was also reported, which could alter cAMP-PDE compartmentation [118].

Within the LV, apex-to-base differences in β_2_AR signalling have been identified. Despite similar cytosolic cAMP levels, it was shown that apical cardiomyocytes exhibit greater contractility in response to β_2_AR stimulation than basal cells. This effect was attributed to enhanced membrane organisation and higher PDE4 activity in basal cardiomyocytes, which restricts cAMP diffusion and modulates β_2_AR signalling [114]. Subcellular compartmentation is critical in β_2_AR signalling, mediated by PDE4 and other proteins. It was demonstrated that β_2_AR localises predominantly to caveolae, invaginations of the sarcolemma enriched in caveolin-3 [119]. Basal cardiomyocytes, which have a higher density of caveolae, exhibit tighter control over cAMP signalling than apical cells [120]. This organisation enables precise spatial regulation of cAMP, contributing to regional functional differences [114]. Moreover, studies using FRET-based sensors showed that PDE4 activity preferentially restricts β_2_AR-associated cAMP signalling to specific microdomains, such as the nucleus, further emphasising the importance of subcellular localisation in PDE4 function [7,114,118,121]. Finally, differences in PDE4 activity across species, including humans and rodents, may reflect evolutionary adaptations to distinct cardiac demands. Such variations underscore the need to carefully interpret animal studies when exploring PDE4 as a human therapeutic target.

### 2.3. PDE4 Isoform-Specific Functions and Subcellular Localisation

PDE4 is the primary regulator of localised cAMP signalling in cardiomyocytes [6,13,46]. Each PDE4 isoform operates in different subcellular microdomains, producing specific effects on cAMP behaviour and subsequent signalling [6,46,114,118]. The unique roles of these isoforms present potential therapeutic targets, especially regarding cardiac physiology.

Below, the specific subcellular localisation of cAMP by PDE4 will be discussed, including its role in β-adrenergic signalling and calcium current regulation, its role in the sarcoplasmic reticulum and signalling at the nuclear envelope. Each of these compartments provides a unique environment where PDE4 finetunes cAMP dynamics to maintain cardiac function.

#### 2.3.1. Plasma Membrane: Modulation of βAR Signalling

At the plasma membrane, various splicing variants of PDE4D interact with β_1_AR and β_2_AR, either directly or via β-arrestin. These interactions modulate the receptors’ sensitivity to ligand stimulation and regulate downstream signalling pathways, including those mediated by other G-protein-coupled receptors (GPCRs). For example, the ability of β-arrestin to scaffold PDE4D ensures that localised cAMP signalling is tightly regulated, which can indirectly influence the activity of other GPCRs sharing overlapping signalling pathways [68,70,72]. This mechanism highlights the importance of PDE4D in maintaining specificity within complex cAMP signalling networks.

In neonatal mouse cardiomyocytes, PDE4D demonstrates isoform-specific regulation of β_2_AR signalling. Notably, PDE4D selectively regulates β_2_AR without affecting β_1_AR signalling, emphasising its role in localising and fine-tuning cAMP signals [122]. It was also shown that PDE4A plays a secondary role in the regulation of βAR signalling in neonatal mouse cardiomyocytes [122]. In PDE4A-KO mice myocytes, isoproterenol-stimulated contraction remains similar to wild types; however, residual PDE4 activity inhibition with rolipram enhances contraction [122].

Ontogenetic studies on the chick ventricular myocardium further support the role of PDE4 in developmental β-adrenergic modulation. Early-stage chick hearts primarily express a Ca^2^⁺/calmodulin-sensitive PDE with characteristics consistent with PDE1C [123], which regulates cAMP and cGMP signalling in human hearts [59]. However, at the late embryonic stage (18E), an additional PDE activity peak emerges, resembling PDE4 based on its substrate specificity and elution profile [123]. This transition coincides with a well-documented 10-fold reduction in β-adrenergic sensitivity of cardiac contraction between embryonic day 16 and hatching (21E), a period marked by the onset of adrenergic neuroeffector transmission in the right ventricle [124]. The emergence of PDE4 at this stage suggests a protective role in limiting excessive β-adrenergic stimulation as sympathetic innervation begins, mirroring its function in mature mammalian hearts, where it prevents cAMP overaccumulation and downstream hyperactivation.

Furthermore, the recruitment of PDE4D5 to β_2_AR by β-arrestin is critical for attenuating pro-hypertrophic signalling pathways. This process involves the inhibition of exchange protein directly activated by cAMP 1 (EPAC1) and calcium/calmodulin-dependent protein kinase II (CaMKII), key mediators of β_2_AR-induced hypertrophic responses [125].

PDE4B and PDE4D isoforms regulate cAMP at the caveolin-rich membranes of rodent cardiomyocytes, with PDE4D predominantly associated with β_2_AR signalling and PDE4B with β_1_AR signalling [48,105,114,126]. PDE4B plays a critical role in modulating cAMP levels near Ryanodine Receptor 2 (RyR2) and negatively regulates LTCC activity, thereby reducing βAR-stimulated ICa,L [48,127]. The loss of PDE4B in neonatal mouse cardiomyocytes results in elevated cAMP at the sarcolemma, enhancing the activity of key proteins like CaV1.2 and RyR2, which are essential for cardiac contraction [105]. In contrast, PDE4D activity is more closely linked to cAMP regulation at the SERCA2a and PLN, influencing calcium cycling [126,127]. Unlike PDE4B and PDE4D, PDE4A does not associate with the LTCC and has no significant impact on β-AR-stimulated ICa,L. Patch-clamp studies show no changes in ICa,L potentiation compared to wild-type mice, suggesting that PDE4A does not directly regulate cardiac calcium signalling [48]. Consistent with this, in PDE4b–/– mice, but not in PDE4a–/– mice, the β-AR response of ICa,L increased, along with an enhancement in cell contraction and Ca^2^⁺ transients, further supporting the specific role of PDE4B in modulating ICa,L and β-AR signalling [48]. The precise localisation of these isoforms underscores their distinct and complementary roles in cardiomyocyte function.

#### 2.3.2. Calcium Current Regulation by PDE4

PDE4 enzymes play a crucial role in modulating calcium dynamics and influencing the susceptibility to arrhythmias in cardiomyocytes [71,72,93]. One research revealed that the inhibition of PDE4 elevates cAMP and enhances calcium currents in human atrial myocytes, leading to increased spontaneous calcium release and a heightened risk of arrhythmias during β-adrenergic stimulation [72]. They also identified that PDE4 activity diminishes with advancing age and experiences a further decline in patients with AF, thereby linking PDE4 dysfunction to increased arrhythmic vulnerability. Additionally, it was demonstrated that the regulatory function of PDE4 becomes particularly significant under conditions of elevated cAMP in human and rabbit atrial cardiomyocytes, notably during β-adrenergic stimulation or the inhibition of PDE3, thereby highlighting its essential role in maintaining calcium homeostasis during periods of physiological stress [71].

The crucial role of PDE4 in regulating the cAMP signalling pathway within adult rat ventricular myocytes was discovered. PDE4 primarily modulates cAMP signalling associated with Gαs-coupled glutamate receptors (Glu-R), which enhances the β_1_AR-mediated increase in L-type Calcium current (ICa,L). Specifically, PDE4 amplifies the Glu-R-induced elevation in cAMP levels, leading to greater stimulation of ICa,L, thereby potentiating β_1_AR responses. While both PDE4 and PDE3 collaborate to regulate β_1_AR and β_2_AR responses, PDE4 becomes the dominant regulator when PDE3 activity is reduced. Furthermore, PDE4 restricts the diffusion of cAMP generated by prostaglandin E1 receptor (PGE1-R) signalling, although this pathway does not significantly influence ICa,L [128].

The importance of PDE4 in calcium regulation is further highlighted by its efficacy in modulating ICa. It was identified that PDE3 and PDE4 are the predominant PDEs involved in regulating basal ICa. By utilising selective PDE inhibitors in isolated rat ventricular myocytes, they revealed that PDE4 plays a dominant role in controlling cAMP levels near LTCC. Upon stimulation, which increases cAMP production, all four PDE subtypes contribute to the calcium current response, exhibiting a clear hierarchy of potency: PDE4 > PDE3 > PDE2 > PDE1 [129]. This hierarchical regulation indicates the critical role of PDE4 in adjusting cellular calcium dynamics in response to diverse signalling inputs.

In experiments with PDE4D−/− mice, increased contractility was observed at baseline and during β-adrenergic stimulation, accompanied by enhanced calcium transients and SR calcium content without changes in ICa,L [130]. These results demonstrate that PDE4D specifically modulates SR calcium cycling, ensuring balanced calcium release and reuptake. Collectively, these findings highlight PDE4’s critical function in protecting against calcium dysregulation, particularly under conditions of stress or disease.

#### 2.3.3. Sarcoplasmic Reticulum: Local Regulation of cAMP Microdomains

PDE4 plays a critical role in regulating calcium dynamics within the sarcoplasmic reticulum (SR). It was demonstrated that inhibiting PDE4 with Ro 20-1724 enhances calcium loading in the SR while simultaneously promoting pro-arrhythmic calcium leaks via PKA and calcium/calmodulin-dependent protein kinase II (CaMKII) pathways in isolated rat cardiomyocytes. Interestingly, the pro-arrhythmic risk could be mitigated through CaMKII inhibition, which preserved positive inotropic effects [113]. These findings underscore the importance of precisely modulating PDE4 activity to balance inotropic benefits against arrhythmogenic risks.

Within the SR, PDE4D is associated with the SERCA2–PLN complex, where it plays a key role in controlling cAMP levels. Reduced PDE4 activity near SERCA2, as observed in mouse hypertrophied cardiomyocytes, can enhance calcium reuptake by increasing PLN phosphorylation, offering potential compensatory benefits in heart failure [131,132,133,134]. However, the implications of PDE4’s role in SR cAMP microdomains are context-dependent, with both beneficial and detrimental effects observed depending on the target and pathological state.

PDE4D3, a specific isoform of PDE4D, is localised within the RyR2 macromolecular complex in the SR. In human heart failure, the association between PDE4D3 and RyR2 is diminished, contributing to hyperphosphorylated and ‘leaky’ RyR2 channels [64]. This pathogenic mechanism is further highlighted by studies in PDE4D-deficient mice, where PKA hyperphosphorylation of RyR2 leads to increased susceptibility to exercise-induced arrhythmias and late-onset dilated cardiomyopathy [93]. Recent investigations using transgenic mice expressing RyR2-targeted cAMP biosensors revealed reduced RyR2-associated PDE4 in hypertrophic conditions, resulting in elevated RyR2 phosphorylation in response to β_2_AR stimulation [73].

#### 2.3.4. Nuclear Envelope

PDE4 enzymes not only regulate calcium dynamics but also play a pivotal role in localised cAMP signalling at the nuclear envelope, where more than half of total PDE4 hydrolytic activity in adult cardiomyocytes occurs [135]. This nuclear compartmentalisation highlights the enzyme’s involvement in transcriptional regulation and hypertrophic signalling pathways. In cardiomyocytes of PDE4d−/− mice, the absence of PDE4D enhances nuclear PKA responses to βAR stimulation, contributing to late-onset dilated cardiomyopathy [136]. These findings link nuclear PDE4D activity to the prevention of pathological cAMP-mediated responses under stress.

Mechanistically, the overexpression of the C-terminal segment of the UCR1 domain (UCR1C) in long-PDE4 isoforms has been shown to inhibit nuclear PKA activity, reducing phosphorylation of the cAMP-responsive element-binding protein (CREB) transcription factor. This suppression of CREB phosphorylation mitigates cardiomyocyte hypertrophy [137]. Furthermore, PDE4D regulates a specific nuclear cAMP pool responsible for PKA-mediated phosphorylation of HSP20, a protein whose phosphorylation provides cardioprotective effects against hypertrophic stimuli [74]. Collectively, these studies illustrate how nuclear PDE4 activity integrates with broader cAMP signalling to protect cardiomyocytes from maladaptive hypertrophic responses.

## 3. A-Kinase-Anchoring Proteins in Cardiomyocytes: Regulators of cAMP Compartmentation and Signalling

### 3.1. Overview of AKAPs: Precision in PKA Signalling

AKAPs are a family of structurally diverse proteins that spatially organise cAMP-dependent PKA signalling activity. Most AKAPs tether the type II PKA holoenzyme to specific subcellular structures through their conserved RII-binding domains [138]. However, it has been shown that AKAP1 can also anchor type I PKA, demonstrating functional diversity among AKAPs [139,140]. AKAPs contain unique targeting domains that localise the AKAP/PKA complex to specific intracellular sites, enabling precise phosphorylation of nearby substrates and maintenance of cAMP/PKA signalling specificity and efficiency [138].

The nomenclature of AKAPs remains complex, as historically, multiple names may refer to the same AKAP discovered by multiple groups. This is summarised in Table 3, which presents the various aliases, key functions and supporting references for each AKAP.

Conventional AKAPs regulate PKA signalling in distinct cardiomyocyte compartments, demonstrating their importance in maintaining compartmentalised cAMP/PKA responses essential for cardiac function [144,145,150,155,174]. However, recent studies have identified non-conventional AKAPs that expand the functional repertoire of anchoring proteins, introducing additional layers of complexity to cardiac signalling.

### 3.2. Non-Conventional AKAPs in Cardiac Signalling

Non-conventional AKAPs are a group of anchoring proteins that go beyond the classical role of tethering PKA to specific subcellular locations. Unlike classical AKAPs, non-conventional AKAPs, such as phosphoinositide 3-kinase gamma (PI3Kγ) and talin, provide dynamic scaffolding structures to their cellular environments, thereby promoting increased regulatory complexity in signalling events [175,176,177,178,179].

PI3Kγ, a class IB PI3K, acts both as a kinase, catalysing the production of phosphatidylinositol (3,4,5)-trisphosphate (PIP3), and as a scaffold that anchors PKA and PDEs to specific signalling microdomains [176,177,178]. Anchored PKA activates PDE to enhance cAMP degradation and phosphorylates p110γ to inhibit PIP3 production [175]. Studies using kinase-dead and -deficient mouse models have revealed distinct roles for PI3Kγ. In kinase-dead mice, the ability to regulate cAMP in specific cellular regions is preserved because the scaffold function of PI3Kγ is intact, while its kinase activity is absent. In contrast, kinase-deficient mice lack both scaffold and kinase functions and display excessive cAMP accumulation and aberrant β-adrenergic signalling, leading to disrupted calcium handling and contractility [177,178]. Thus, PI3Kγ plays a dual role in cardiomyocyte β-adrenergic signalling, where it regulates cAMP dynamics through its scaffold function while influencing downstream pathways, such as Akt signalling, through its kinase activity [180,181].

Talin, a focal adhesion protein, represents another non-conventional AKAP with its function tightly linked to mechanotransduction [182,183]. Talin bridges integrins and the actin cytoskeleton, forming a mechanically sensitive scaffold that responds to cellular tension [182]. Recent studies have demonstrated that talin binds PKA in a force-dependent manner, working as a mechanically gated AKAP [179]. Under mechanical stress, talin’s R9 domain unfolds, exposing a cryptic PKA-binding site. This interaction anchors PKA to focal adhesions and enables the phosphorylation of targets such as Vasodilator-Stimulated Phosphoprotein, a key regulator of actin dynamics. The talin–PKA complex illustrates a new paradigm of signal regulation, where mechanotransduction directly influences the spatial and temporal activity of PKA [179].

Non-conventional AKAPs, such as PI3Kγ and talin, extend the regulatory scope of cAMP/PKA signalling in cardiomyocytes [176,177,178,179]. PI3Kγ anchors PDE4 to βAR microdomains, regulating cAMP levels and calcium homeostasis [176,177,178]. While talin’s direct role in PDE4 regulation remains unstudied, its classification as an AKAP suggests potential involvement in cAMP signalling pathways [179].

PI3Kγ and talin underscore the importance of non-conventional AKAPs in fine-tuning cAMP signalling, ensuring calcium homeostasis, contractility and protection against cardiac remodelling.

### 3.3. AKAP-PDE4-PKA Complexes: Masters of cAMP Regulation

PDE4D3 emerges as a major regulator of localised cAMP dynamics through anchoring various AKAPs, including AKAP9, mAKAP, and AKAP12 [106,184,185,186].

Several works emphasise the importance of the complex between mAKAP and PDE4D3 [186]. mAKAP establishes a critical scaffold at the nuclear envelope of cardiomyocytes, orchestrating a tightly regulated cAMP signalling network essential for maintaining cardiac homeostasis. The basis of this network is PDE4D3, which binds to mAKAP and acts as a negative feedback regulator by degrading cAMP generated in response to adrenergic stimulation [186]. This localised cAMP control is enhanced by PKA, also anchored by mAKAP, which phosphorylates PDE4D3 at Ser-13 and Ser-54, strengthening its enzymatic activity and ensuring precise spatial and temporal modulation of cAMP signalling [187,188].

At the SR, mAKAP is directly associated with RyR through a conserved leucine zipper motif, forming a key signalling complex that regulates calcium dynamics [189]. This localisation facilitates PKA-mediated phosphorylation of RyR at Ser-2808, increasing its open probability and calcium sensitivity. However, in pathological states such as heart failure, reduced PDE4D3 levels in the mAKAP complex lead to RyR hyperphosphorylation, contributing to calcium leakage from the SR and promoting arrhythmias and cardiac dysfunction [93]. These findings illustrate how the mAKAP–PDE4D3 complex integrates cAMP and calcium signalling, influencing both nuclear and cytoplasmic processes, with critical implications for cardiomyocyte function and maladaptive remodelling.

Within cardiac myocytes, PDE4D3 associates with the IKs potassium channel complex via AKAP9, ensuring the precise modulation of channel activity under adrenergic stimulation [184]. Similarly, AKAP12 anchors PDE4D3 and PKA to subplasmalemmal domains, enabling the rapid degradation of cAMP and maintaining signalling accuracy at the plasma membrane [106]. Interestingly, AKAP12 anchors PDE8A in a similar way [53,100]. The disruption of both complexes results in dysregulated cAMP gradients, with prolonged signalling evident upon AKAP12 knockdown [106,184]. Altogether, these studies underscore the critical role of AKAP-anchored PDE4D3 in the organisation of cAMP compartmentation and supporting cellular homeostasis.

PI3Kγ, as a non-conventional AKAP, can form complexes with different PDEs [175,176,177]. PI3Kγ anchors PKA alongside PDE isoforms, particularly PDE4A, PDE4B and PDE3A in cardiomyocytes. These PI3Kγ-regulated PDEs reduce cAMP levels, thereby restricting PKA-mediated phosphorylation of the LTCC and PLN, ensuring the precise control of calcium handling and cardiac contractility [177]. In the absence of PI3Kγ, PDE4 activity is impaired, resulting in the hyperphosphorylation of calcium-handling proteins and arrhythmic calcium transients [177]. Furthermore, pharmacological studies have revealed that targeting the PKA-PDE4B/PDE4D complexes mediated by PI3Kγ could offer therapeutic strategies to elevate cAMP in chronic obstructive respiratory diseases [77,176].

AKAP–PDE4–PKA complexes are essential for compartmentalised cAMP signalling, enabling the precise regulation of calcium dynamics, contractility and membrane excitability in cardiomyocytes [93,106,184,185,186,189]. Examples such as the mAKAP-PDE4D3 scaffold at the nuclear envelope and SR and PI3Kγ–PDE complexes underscore the critical role of localised cAMP control in maintaining cardiac homeostasis [106,184,185,186]. Dysregulation of these complexes has been implicated in arrhythmias, heart failure and maladaptive remodelling, making them promising therapeutic targets [93,189]. However, further research is needed to fully understand their mechanisms and evaluate clinical applicability, paving the way for targeted therapies addressing cAMP-driven cardiac pathologies.

## 4. PDE4 and AKAPs in Heart Failure: Implications for cAMP Signalling and Cardiac Remodelling

### 4.1. PDE4 and Heart Failure

Heart failure and cardiac hypertrophy are characterised by significant alterations in the expression and activity of PDE4 in cardiomyocytes, which disrupts cAMP signalling and contributes to maladaptive remodelling. A decrease in the activity of PDE4A and PDE4D has been observed in failing hearts [47], while compartment-specific alterations in the activity of PDE4 and PDE3 have been reported in hypertrophic ventricular myocytes [190]. Chronic exposure to catecholamines leads to a reduction in PDE4 and PDE3 activity in PKA-RI compartments while simultaneously enhancing PDE4 activity in PKA-RII compartments, thereby exacerbating pathological remodelling. The observed shift in the equilibrium between PDE3 and PDE4 favours PDE3 in pathological conditions, diminishing the regulatory influence of PDE4 on cAMP levels [191,192]. PKA-RI is predominantly found in cytosolic regions and responds rapidly to transient cAMP increases. In contrast, PKA-RII is enriched in subcellular structures, including the sarcoplasmic reticulum and nuclear envelope, enabling sustained and localised cAMP signalling [126,193]. Interestingly, in a rat model of cardiac hypertrophy induced by thoracic aortic banding, the total cAMP hydrolytic activity and activities of PDE4 and PDE3 isoforms—including PDE4A and PDE4B—are reduced, whereas PDE4D activity remains unchanged [191]. These findings suggest that PDE4A may play a role in pathological remodelling during heart failure, potentially through its contribution to broader changes in cAMP signalling dynamics or the synergistic effect of cAMP inhibition.

Moreover, the redistribution of β_2_ARs and the alteration of cAMP compartmentation reduce the protective mechanisms afforded by β_2_ARs, resulting in desensitisation and compromised cardiac function [194]. The restoration of PDE4B activity through transgenic overexpression or adeno-associated virus serotype 9 (AAV9) gene therapy alleviates pathological remodelling and normalises cAMP signalling, highlighting the therapeutic potential of PDE4 targeting [110]. The overexpression of PDE4B3 reduces cardiac hypertrophy and fibrosis while restoring cAMP compartmentation in RyR and caveolin-rich microdomains. This therapy also decreases RyR2 phosphorylation and arrhythmic events, showing potential in patient-derived cardiomyocytes with specific genetic mutations. These findings suggest that PDE4B3 modulation could offer targeted treatment for heart failure and associated arrhythmias [194]. Furthermore, it was reported that the deletion of PDE4D leads to an increase in RyR2 phosphorylation and an elevated risk of arrhythmias, thereby further accelerating the progression of heart failure [93]. Altogether, these findings highlight the key role of PDE4 isoforms in the maintenance of cardiac function and their potential as therapeutic targets in the context of heart failure.

Targeting PDE4 isoforms presents a promising treatment strategy for heart failure by restoring cAMP compartmentation and calcium dynamics while mitigating the adverse effects of prolonged catecholamine stimulation. Isoform-specific modulation, such as increasing PDE4 activity in protective microdomains (e.g., SERCA2 and the nuclear envelope) or blocking pro-arrhythmic pathways driven by PKA and CaMKII, may tackle significant pathological mechanisms. Innovative strategies, such as gene therapy and pharmacological agents, present chances to utilise the therapeutic potential of PDE4 to diminish maladaptive cardiac remodelling and enhance results in heart failure.

### 4.2. AKAPs in Heart Failure: Mechanistic Roles and Pathological Impact

AKAPs are essential in the spatial and temporal regulation of signalling pathways critical for cardiac physiology and pathology [145,150,155,173,177]. AKAP1 exerts an anti-hypertrophic effect by sequestering calcineurin, thereby inhibiting NFAT-dependent transcriptional programs that can lead to pathological cardiomyocyte growth. The knockdown of AKAP121 leads to hypertrophy in neonatal rat cardiomyocytes, whereas its overexpression suppresses isoproterenol-induced hypertrophic responses, positioning it as a potential therapeutic target for calcineurin/NFAT modulation [195]. Similarly, AKAP13 acts as a scaffold for protein kinase D (PKD), coordinating the phosphorylation and nuclear export of HDAC5 to relieve MEF2 repression and promote hypertrophic gene expression. Disrupting the PKD binding site on AKAP13 decreases hypertrophic responses, emphasising its role in pathological remodelling [172].

The nuclear envelope-anchored AKAP6 integrates diverse hypertrophic signals, coordinating transcriptional regulation through interactions with PLCε [196], PKD [197] and various transcription factors, including MEF2 [197,198] and NFAT [197]. These complexes facilitate the precise control of local signalling, such as PKD-mediated HDAC phosphorylation, driving hypertrophic remodelling [197]. The loss of AKAP6, a scaffold that coordinates key signalling pathways at the nuclear envelope, reduces pressure-overload-induced hypertrophy, fibrosis and apoptosis, as shown by improved survival and attenuated remodelling in AKAP6-deficient mice [196,197,199]. Furthermore, alterations in AKAP-mediated cAMP dynamics are evident in heart failure models. For example, AKAP7 and PDE4 regulate β_2_AR signalling in the PLN/SERCA2a microdomain. Dysregulation of this axis in HFpEF due to obesity and diabetes leads to enhanced β_2_AR-mediated cAMP production, PLN phosphorylation and calcium reuptake, a compensatory mechanism that may contribute to maladaptive remodelling [200].

Together, these findings underscore the complexity and therapeutic promise of targeting AKAPs to modulate maladaptive cardiac remodelling and improve outcomes in heart failure.

## 5. Therapeutical Insights and Future Perspectives

Efforts to refine PDE4 inhibitors have been ongoing for decades, aiming to enhance selectivity, minimise side effects and expand their therapeutic potential [201,202,203,204,205,206]. These advancements have led to the development of several PDE4 inhibitors, including roflumilast, apremilast and crisaborole, which have completed Phase IV clinical trials. Additionally, other drugs—such as Hemay005, cilomilast and tanimilas—are in various stages of clinical development, with some still undergoing Phase III trials while others have progressed further for indications ranging from chronic respiratory diseases to inflammatory and dermatological conditions (Table 4) [202,207,208,209]. Further advancements in PDE4 inhibitor development have driven the need for greater specificity to enhance efficacy and reduce adverse effects. This has led to the emergence of targeted PDE4 inhibitors, such as GSK256066 (selective for PDE4B) [210] and BPN14770 (selective for PDE4D) [205], which are currently in phase II clinical trials for Chronic Obstructive Pulmonary Disease (COPD) and Fragile X Syndrome, respectively (Table 4). Currently, KIT2014, a PI3Kγ-mimetic peptide that inhibits PDE4B and PDE4D [176], is undergoing a phase I clinical trial as a potential alternative to classical PDE4 inhibitors (Table 4).

Roflumilast, a pan-PDE4 inhibitor approved in 2010 for severe chronic obstructive pulmonary disease, exerts its effects via its active metabolite, roflumilast-N-oxide [208]. Importantly, pooled clinical trial data have demonstrated that roflumilast does not increase cardiovascular risk, with rates of major adverse cardiovascular events (MACEs) remaining low and comparable to placebo [212]. This favourable safety profile is particularly relevant given the potential for PDE4 inhibitors to influence cardiovascular physiology. Similarly, apremilast, introduced in 2014, is a pan-PDE4 inhibitor initially approved for psoriasis and later for psoriatic arthritis and Behçet’s disease. Despite its lack of isoform specificity, apremilast has demonstrated significant efficacy and tolerability in treating inflammatory conditions, making it a valuable therapeutic option [209].

Recognising the need for greater isoform specificity, researchers have shifted their focus to developing inhibitors targeting specific PDE4 isoforms, particularly PDE4B and PDE4D, which hold promise in cardiac disease management. In the heart, localised distortions of PDE activity are implicated in several pathological conditions. For example, maladaptive remodelling in heart failure is associated with altered cAMP degradation within key microdomains, such as those around the SR and LTCC. Reduced cAMP levels and impaired PKA activity in these regions disrupt calcium handling and contractility, contributing to disease progression [47,93,189,190]. In arrhythmias, increased PDE activity within RyR2 microdomains leads to inadequate cAMP signalling, promoting calcium leaks and electrical instability [71,72,93]. These findings underscore the importance of precisely targeting specific PDE4 isoforms within distinct subcellular domains to restore localised cAMP dynamics without systemic disruption.

Novel PDE4D allosteric modulators were developed that selectively interact with the enzyme’s UCR2 regulatory domain. Unlike traditional active site inhibitors that fully suppress activity, these modulators partially inhibit enzymatic activity, preserving the spatial and temporal control of cAMP signalling. This selective inhibition reduces adverse effects, such as emesis, a common limitation of pan-PDE4 inhibitors. Co-crystal structural studies revealed how these modulators stabilise the UCR2 domain in a “closed” conformation, effectively capping the catalytic domain to modulate activity [206]. Similarly, PDE4B/D-selective inhibitors have shown improved selectivity and reduced toxicity [211,213], further supporting their potential in cardiac applications. Recent advances in PROteolysis-TArgeting Chimera (PROTAC) technology offer a novel approach to PDE4 modulation. For instance, the BI 1015550-based PROTAC, KTX207, selectively targets PDE4D shortforms with remarkable potency, achieving an IC50 of approximately 10 pM for degradation. This strategy enhances efficacy in suppressing inflammatory markers and promises a superior side effect profile compared to conventional catalytic site inhibitors, making it a promising tool for precise cAMP regulation in cardiac applications [214].

Targeting PDE4 isoforms within specific cardiac microdomains offers a promising therapeutic strategy for conditions like heart failure and arrhythmias. By restoring localised cAMP signalling, these approaches may address pathological remodelling and improve cardiac function while minimising systemic toxicity. Continued research into isoform-selective PDE4 inhibitors could pave the way for precision therapies that balance efficacy and safety in cardiovascular diseases.

Alternative future therapeutic strategies for cardiac diseases could involve combining isoform-specific PDE4 inhibitors with precision targeting of their interacting partners, such as AKAPs and POPDC1, to enhance localised cAMP regulation within key cardiac microdomains. This approach could help restore cAMP dynamics in pathological conditions like heart failure and arrhythmias, where disrupted compartmentalisation contributes to maladaptive remodelling and impaired contractility. Advances in gene therapy and mRNA-based technologies offer additional potential by enabling the precise modulation of specific PDE4 isoforms in subcellular domains such as the sarcoplasmic reticulum and plasma membrane. These strategies could directly address local cAMP deficiencies, reduce maladaptive remodelling and improve calcium handling and overall cardiac function. Furthermore, high-throughput screening platforms and computational models could aid in the discovery of novel allosteric modulators or small molecules, paving the way for safer and more effective PDE4-based therapies tailored to individual patient needs.

## 6. Conclusions: Can PDE4 Still Be Considered Only a Minor Helper in the Heart?

The PDE4 family is essential in regulating cardiomyocyte physiology and pathophysiology, intricately balancing localised cAMP signalling [6,7,67,71,72,93]. In the heart, the PDE4A, PDE4B and PDE4D isoforms function in distinct subcellular microdomains, affecting cardiac contractility, calcium handling and hypertrophic responses often via complex formation with other regulatory and effector proteins [48,105,114,126]. These complexes maintain homeostasis and critical regulators during normal physiological conditions, while their dysfunction can contribute to severe pathological states, including heart failure and arrhythmias [48,106,122,184].

Species-specific and regional variations in PDE4 activity underscore the complexity of translating rodent-based findings to human cardiology [47,72,93,94,112]. For instance, while PDE4 plays a minor role in overall cAMP hydrolysis in human hearts [94,112], it is crucial for compartmentalised signalling, particularly under β-adrenergic stimulation [68,69,70,114]. The cooperative dynamics between PDE4 and PDE3 further emphasise the necessity of a deep understanding of their roles in modulating cardiac responses, especially in the disease context.

Therapeutic strategies targeting PDE4 have evolved significantly, moving from broad-spectrum inhibitors to isoform-specific modulators. Advances in selective PDE4 inhibitors, such as those targeting PDE4B and PDE4D, offer promising strategies for minimising systemic toxicity while enhancing therapeutic efficacy [211,213]. Additionally, the selective inhibition of specific PDE pools through AKAP-disrupting peptides, such as PI3Kγ mimetic peptides, presents another targeted approach for modulating compartmentalised cAMP signalling [176,215,216]. These innovations are particularly relevant for treating heart failure, where maladaptive remodelling and arrhythmogenesis demand precise modulation of cAMP signalling.

Future research should prioritise the development of isoform-specific therapies that will allow for more fine-tuning regulation of cAMP. Gene therapies, allosteric modulators and combination strategies targeting PDE4’s interactions with key proteins like AKAPs and POPDC1 can potentially transform clinical approaches. Furthermore, these therapies must be designed with an acute awareness of species differences to ensure successful translation from bench to bedside.

By bridging fundamental molecular insights with clinical applications, studying the PDE4 family opens pathways to novel treatments that restore cardiac function, mitigate heart failure progression and offer more targeted, safer therapeutic approaches.

## Figures and Tables

**Table 1:** Classification of phosphodiesterase families based on substrate specificity and isoforms.

Category	PDE Family	Isoforms	References
cAMP-specific PDEs	PDE4	PDE4A, PDE4B, PDE4C, PDE4D	[14,15]
	PDE7	PDE7A, PDE7B	[16,17]
	PDE8	PDE8A, PDE8B	[18,19,20]
cGMP-specific PDEs	PDE5	PDE5A	[21,22,23]
	PDE6	PDE6A, PDE6B, PDE6C	[24,25]
	PDE9	PDE9A	[26,27,28]
Dual-substrate PDEs	PDE1	PDE1A, PDE1B, PDE1C	[29,30,31]
	PDE2	PDE2A	[32]
	PDE3	PDE3A, PDE3B	[33,34,35,36]
	PDE10	PDE10A	[37,38]
	PDE11	PDE11A	[39,40]

**Table 2:** Enzymatic properties and substrate specificity of cardiac PDEs.

Category	PDE Family	Main Functions and Localisation	Km (μmol/l)		References
			cAMP	cGMP	
cAMP-specific PDEs	PDE4	Plays a key role when cAMP levels are elevated. Detected in cardiomyocytes and fibroblasts.	1–6	NA	[15,47,48,49,50,51,52]
	PDE8	Controls ICa,L current. Detected in cardiomyocytes.	0.1–0.6	NA	[19,20,51,53,54]
cGMP-specific PDEs	PDE5	Preferentially regulates a pool of cGMP produced by soluble GC. Detected in cardiomyocytes and fibroblasts.	201	1–6	[2,21,22,23,51,55,56]
	PDE9	Preferentially regulates the NP-induced cGMP. Detected in cardiomyocytes and not detected in fibroblasts.	230	0.1–0.4	[2,26,27,28,51,57,58]
Dual-substrate PDEs	PDE1	Regulation of calcium/calmodulin. Detected in cardiomyocytes and fibroblasts.	1–125	1–8	[29,51,59,60]
	PDE2	Regulates local mitochondria-related cAMP pools. More abundantly expressed in cardiac fibroblasts compared to cardiomyocytes.	30–112	10–31	[6,51,61,62]
	PDE3	Responsible for the tonic effects in the myocardium. PDE3 is the most important in cardiomyocytes.	0.1–0.8	0.1–0.8	[33,34,36,60,63,64,65]
	PDE10	cAMP regulates PDE10 biphasically, modulating cGMP hydrolysis. Detected in cardiomyocytes and fibroblasts.	0.2–0.3	1.1–7.2	[38,45,51,66]

**Table 3:** AKAP isoforms in cardiomyocyte regulation: key roles and references.

AKAP	Aliases	Functions in Cardiomyocytes
AKAP1	D-AKAP1 [139], AKAP121 [141], AKAP149 [142], S-AKAP84 [143], mitoAKAP [144]	Regulates mitochondrial dynamics, oxidative phosphorylation, and cardiomyocyte survival, playing a protective role against cardiac hypertrophy and heart failure [144,145].
AKAP2	D-AKAP2 [140]	Organises a signalling complex with PKA and Src3, promoting anti-apoptotic and pro-angiogenic responses essential for myocardial infarction recovery [146].
AKAP5	AKAP79 [147], AKAP150 [148], AKAP75 [149]	Coordinates PKA signalling in T-tubules and plasma membrane, regulating calcium channels and cardiac contractility under sympathetic stimulation [150,151].
AKAP6	mAKAPβ [152], AKAP100 [138], mAKAP [153]	Regulates calcium handling by interacting with PLN and organises the nuclear envelope microtubule-organisng centre through centrosomal and Golgi-associated proteins [154,155].
AKAP7	AKAP15 [156], AKAP18 [157]	Localises PKA to the plasma membrane, regulating membrane events like cardiac IK1 currents [158,159,160].
AKAP9	Yotiao [161], AKAP350 [162], AKAP450 [163]	Coordinates β-adrenergic regulation of the IKs potassium channel by assembling PKA, PP1, AC9, and PDE4D3 into a macromolecular complex. Disruptions are linked to long-QT syndrome and impaired cardiac repolarisation [164,165,166].
AKAP12	Gravin [167], SSeCKS [168], AKAP250 [169]	Mitigates maladaptive remodelling, oxidative stress, and fibrosis by inhibiting Ang-II-induced TGFβ1 signalling. Also regulates cardiac contractility and calcium handling during isoproterenol stimulation [53,170].
AKAP13	AKAP-Lbc [171]	Coordinates cardiomyocyte signalling pathways involved in protection against doxorubicin toxicity, pathological hypertrophy, and α1-adrenergic receptor-mediated RhoA activation [172,173,174].

**Table 4:** PDE4 inhibitors: specificity, clinical indications and trial phases.

Drug Name	PDE4 Specificity	Disease	Phase	NCT Number *
Roflumilast [208]	Pan-PDE4	Polycystic Ovary Syndrome	IV	NCT02037672; NCT02187250
		Chronic Hand Eczema	IV	NCT05682859
		Ulcerative Colitis	IV	NCT05684484
		Chronic Obstructive Pulmonary Disease	IV	NCT01595750
Apremilast [209]	Pan-PDE4	Recurrent Aphthous Stomatitis (RAS)	IV	NCT03690544
		Alopecia Areata	IV	NCT05926882
		Chronic and Recurrent Erythema Nodosum Leprosum	IV	NCT04822909
		Oral Lichen Planus	IV	NCT06260904
Crisaborole [207]	Pan-PDE4	Moderate Atopic Dermatitis	IV	NCT04214197
		Seborrheic Dermatitis	IV	NCT03567980
Hemay005 [211]	Pan-PDE4	Behçet’s Disease	III	NCT06145893
		Severe Plaque Psoriasis	III	NCT04839328
Cilomilast [208]	Pan-PDE4, more selective for PDE4D	Chronic Obstructive Pulmonary Disease	III	NCT00103922
Tanimilast [211]	Pan-PDE4	Chronic Obstructive Pulmonary Disease and Chronic Bronchitis	III	NCT04636801
GSK256066 [210]	PDE4B	Chronic Obstructive Pulmonary Disease	II	NCT00549679
BPN14770 [205]	PDE4D	Fragile X Syndrome	II	NCT03569631
KIT2014 [176]	PDE4B and PDE4D	Healthy Subjects	I	NCT06659757

* ClinicalTrials.gov identifiers from https://clinicaltrials.gov (accessed on 16 March 2025).

## Data Availability

Not applicable.

## Abbreviations

The following abbreviations are used in this manuscript:

βAR	Beta-Adrenergic Receptor
β_1_AR	Beta-1-Adrenergic Receptor
β_2_AR	Beta-2-Adrenergic Receptor
AKAP	A-Kinase Anchoring Protein
cAMP	Cyclic Adenosine Monophosphate
cGMP	Cyclic Guanosine Monophosphate
CaMKII	Calcium/Calmodulin-Dependent Protein Kinase II
CREB	cAMP Response Element-Binding Protein
ERK2	Extracellular Signal-Regulated Kinase 2
GPCR	G-Protein Coupled Receptor
HFpEF	Heart Failure with Preserved Ejection Fraction
ICa,L	L-Type Calcium Current
LTCC	L-Type Calcium Channel
LV	Left Ventricle
mAKAP	Muscle A-Kinase Anchoring Protein
PDE	Phosphodiesterase
PDE4A, PDE4B, PDE4C, PDE4D	Isoforms of Phosphodiesterase 4
PKA	Protein Kinase A
PKA-RI/RII	Protein Kinase A Regulatory Subunit I/II
PKD	Protein Kinase D
PLN	Phospholamban
POPDC1	Popeye Domain Containing 1
RyR2	Ryanodine Receptor 2
SERCA2a	Sarco/Endoplasmic Reticulum Calcium-ATPase 2a
SR	Sarcoplasmic Reticulum
UCR	Upstream Conserved Region
VASP	Vasodilator-stimulated phosphoprotein

## References

[1] Puertas-Umbert L., Alonso J., Hove-Madsen L., Martínez-González J., Rodríguez C. (2023). PDE4 Phosphodiesterases in Cardiovascular Diseases: Key Pathophysiological Players and Potential Therapeutic Targets. Int. J. Mol. Sci..

[2] Fu Q., Wang Y., Yan C., Xiang Y.K. (2024). Phosphodiesterase in Heart and Vessels: From Physiology to Diseases. Physiol. Rev..

[3] Baillie G.S., Tejeda G.S., Kelly M.P. (2019). Therapeutic Targeting of 3′,5′-Cyclic Nucleotide Phosphodiesterases: Inhibition and Beyond. Nat. Rev. Drug Discov..

[4] Bers D.M., Xiang Y.K., Zaccolo M. (2019). Whole-Cell cAMP and PKA Activity Are Epiphenomena, Nanodomain Signaling Matters. Physiology.

[5] Omori K., Kotera J. (2007). Overview of PDEs and Their Regulation. Circ. Res..

[6] Zaccolo M., Movsesian M.A. (2007). cAMP and cGMP Signaling Cross-Talk: Role of Phosphodiesterases and Implications for Cardiac Pathophysiology. Circ. Res..

[7] Mika D., Leroy J., Vandecasteele G., Fischmeister R. (2012). PDEs Create Local Domains of cAMP Signaling. J. Mol. Cell. Cardiol..

[8] Kim C., Xuong N.-H., Taylor S.S. (2005). Crystal Structure of a Complex Between the Catalytic and Regulatory (RIα) Subunits of PKA. Science.

[9] Viste K., Kopperud R.K., Christensen A.E., Døskeland S.O. (2005). Substrate Enhances the Sensitivity of Type I Protein Kinase A to cAMP. J. Biol. Chem..

[10] Cheadle C., Nesterova M., Watkins T., Barnes K.C., Hall J.C., Rosen A., Becker K.G., Cho-Chung Y.S. (2008). Regulatory Subunits of PKA Define an Axis of Cellular Proliferation/Differentiation in Ovarian Cancer Cells. BMC Med. Genom..

[11] Di Benedetto G., Zoccarato A., Lissandron V., Terrin A., Li X., Houslay M.D., Baillie G.S., Zaccolo M. (2008). Protein Kinase A Type I and Type II Define Distinct Intracellular Signaling Compartments. Circ. Res..

[12] Burgers P.P., Bruystens J., Burnley R.J., Nikolaev V.O., Keshwani M., Wu J., Janssen B.J.C., Taylor S.S., Heck A.J.R., Scholten A. (2016). Structure of Sm AKAP and Its Regulation by PKA -mediated Phosphorylation. FEBS J..

[13] Stangherlin A., Gesellchen F., Zoccarato A., Terrin A., Fields L.A., Berrera M., Surdo N.C., Craig M.A., Smith G., Hamilton G. (2011). cGMP Signals Modulate cAMP Levels in a Compartment-Specific Manner to Regulate Catecholamine-Dependent Signaling in Cardiac Myocytes. Circ. Res..

[14] Manning C.D., Burman M., Christensen S.B., Cieslinski L.B., Essayan D.M., Grous M., Torphy T.J., Barnette M.S. (1999). Suppression of Human Inflammatory Cell Function by Subtype-selective PDE4 Inhibitors Correlates with Inhibition of PDE4A and PDE4B. Br. J. Pharmacol..

[15] Wang P., Myers J.G., Wu P., Cheewatrakoolpong B., Egan R.W., Billah M.M. (1997). Expression, Purification, and Characterization of Human cAMP-Specific Phosphodiesterase (PDE4) Subtypes A, B, C, and D. Biochem. Biophys. Res. Commun..

[16] Han P., Zhu X., Michaeli T. (1997). Alternative Splicing of the High Affinity cAMP-Specific Phosphodiesterase (PDE7A) mRNA in Human Skeletal Muscle and Heart. J. Biol. Chem..

[17] Hetman J.M., Soderling S.H., Glavas N.A., Beavo J.A. (2000). Cloning and Characterization of PDE7B, a cAMP-Specific Phosphodiesterase. Proc. Natl. Acad. Sci. USA.

[18] Fisher D.A., Smith J.F., Pillar J.S., St. Denis S.H., Cheng J.B. (1998). Isolation and Characterization of PDE8A, a Novel Human cAMP-Specific Phosphodiesterase. Biochem. Biophys. Res. Commun..

[19] Soderling S.H., Bayuga S.J., Beavo J.A. (1998). Cloning and Characterization of a cAMP-Specific Cyclic Nucleotide Phosphodiesterase. Proc. Natl. Acad. Sci. USA.

[20] Gamanuma M., Yuasa K., Sasaki T., Sakurai N., Kotera J., Omori K. (2003). Comparison of Enzymatic Characterization and Gene Organization of Cyclic Nucleotide Phosphodiesterase 8 Family in Humans. Cell. Signal..

[21] Loughney K., Hill T.R., Florio V.A., Uher L., Rosman G.J., Wolda S.L., Jones B.A., Howard M.L., McAllister-Lucas L.M., Sonnenburg W.K. (1998). Isolation and Characterization of cDNAs Encoding PDE5A, a Human cGMP-Binding, cGMP-Specific 3′,5′-Cyclic Nucleotide Phosphodiesterase. Gene.

[22] Lin C.-S., Lau A., Tu R., Lue T.F. (2000). Expression of Three Isoforms of cGMP-Binding cGMP-Specific Phosphodiesterase (PDE5) in Human Penile Cavernosum. Biochem. Biophys. Res. Commun..

[23] Wang P., Wu P., Myers J.G., Stamford A., Egan R.W., Billah M.M. (2001). Characterization of Human, Dog and Rabbit Corpus Cavernosum Type 5 Phosphodiesterases. Life Sci..

[24] Gillespie P.G., Beavo J.A. (1988). Characterization of a Bovine Cone Photoreceptor Phosphodiesterase Purified by Cyclic GMP-Sepharose Chromatography. J. Biol. Chem..

[25] Muradov H., Boyd K.K., Artemyev N.O. (2010). Rod Phosphodiesterase-6 PDE6A and PDE6B Subunits Are Enzymatically Equivalent. J. Biol. Chem..

[26] Fisher D.A., Smith J.F., Pillar J.S., Denis S.H.S., Cheng J.B. (1998). Isolation and Characterization of PDE9A, a Novel Human cGMP-Specific Phosphodiesterase. J. Biol. Chem..

[27] Soderling S.H., Bayuga S.J., Beavo J.A. (1998). Identification and Characterization of a Novel Family of Cyclic Nucleotide Phosphodiesterases. J. Biol. Chem..

[28] Wang P., Wu P., Egan R.W., Billah M.M. (2003). Identification and Characterization of a New Human Type 9 cGMP-Specific Phosphodiesterase Splice Variant (PDE9A5). Gene.

[29] Snyder P.B., Florio V.A., Ferguson K., Loughney K. (1999). Isolation, Expression and Analysis of Splice Variants of a Human Ca^2+^/Calmodulin-Stimulated Phosphodiesterase (PDE1A). Cell. Signal..

[30] Sharma R.K., Wang J.H. (1986). Calmodulin and Ca^2+^-Dependent Phosphorylation and Dephosphorylation of 63-kDa Subunit-Containing Bovine Brain Calmodulin-Stimulated Cyclic Nucleotide Phosphodiesterase Isozyme. J. Biol. Chem..

[31] Kincaid R.L., Stith-Coleman I.E., Vaughan M. (1985). Proteolytic Activation of Calmodulin-Dependent Cyclic Nucleotide Phosphodiesterase. J. Biol. Chem..

[32] Rosman G.J., Martins T.J., Sonnenburg W.K., Beavo J.A., Ferguson K., Loughney K. (1997). Isolation and Characterization of Human cDNAs Encoding a cGMP-Stimulated 3′,5′-Cyclic Nucleotide Phosphodiesterase. Gene.

[33] Harrison S.A., Reifsnyder D.H., Gallis B., Cadd G.G., Beavo J.A. (1986). Isolation and Characterization of Bovine Cardiac Muscle cGMP-Inhibited Phosphodiesterase: A Receptor for New Cardiotonic Drugs. Mol. Pharmacol..

[34] Grant P.G., Colman R.W. (1984). Purification and Characterization of a Human Platelet Cyclic Nucleotide Phosphodiesterase. Biochemistry.

[35] Meacci E., Taira M., Moos M., Smith C.J., Movsesian M.A., Degerman E., Belfrage P., Manganiello V. (1992). Molecular Cloning and Expression of Human Myocardial cGMP-Inhibited cAMP Phosphodiesterase. Proc. Natl. Acad. Sci. USA.

[36] Degerman E., Belfrage P., Newman A.H., Rice K.C., Manganiello V.C. (1987). Purification of the Putative Hormone-Sensitive Cyclic AMP Phosphodiesterase from Rat Adipose Tissue Using a Derivative of Cilostamide as a Novel Affinity Ligand. J. Biol. Chem..

[37] Fujishige K., Kotera J., Michibata H., Yuasa K., Takebayashi S., Okumura K., Omori K. (1999). Cloning and Characterization of a Novel Human Phosphodiesterase That Hydrolyzes Both cAMP and cGMP (PDE10A). J. Biol. Chem..

[38] Loughney K., Snyder P.B., Uher L., Rosman G.J., Ferguson K., Florio V.A. (1999). Isolation and Characterization of PDE10A, a Novel Human 3′, 5′-Cyclic Nucleotide Phosphodiesterase. Gene.

[39] Fawcett L., Baxendale R., Stacey P., McGrouther C., Harrow I., Soderling S., Hetman J., Beavo J.A., Phillips S.C. (2000). Molecular Cloning and Characterization of a Distinct Human Phosphodiesterase Gene Family: PDE11A. Proc. Natl. Acad. Sci. USA.

[40] Hetman J.M., Robas N., Baxendale R., Fidock M., Phillips S.C., Soderling S.H., Beavo J.A. (2000). Cloning and Characterization of Two Splice Variants of Human Phosphodiesterase 11A. Proc. Natl. Acad. Sci. USA.

[41] Loughney K., Martins T.J., Harris E.A.S., Sadhu K., Hicks J.B., Sonnenburg W.K., Beavo J.A., Ferguson K. (1996). Isolation and Characterization of cDNAs Corresponding to Two Human Calcium, Calmodulin-Regulated, 3′,5′-Cyclic Nucleotide Phosphodiesterases. J. Biol. Chem..

[42] Kostic M.M., Erdogan S., Rena G., Borchert G., Hoch B., Bartel S., Scotland G., Huston E., Houslay M.D., Krause E.-G. (1997). Altered Expression of PDE1 and PDE4 Cyclic Nucleotide Phosphodiesterase Isoforms in 7-Oxo-Prostacyclin-Preconditioned Rat Heart. J. Mol. Cell. Cardiol..

[43] Senzaki H., Smith C.J., Juang G.J., Isoda T., Mayer S.P., Ohler A., Paolocci N., Tomaselli G.F., Hare J.M., Kass D.A. (2001). Cardiac Phosphodiesterase 5 (cGMP-specific) Modulates Β-adrenergic Signaling in Vivo and Is Down-regulated in Heart Failure. FASEB J..

[44] Ónody A., Zvara Á., Hackler L., Vígh L., Ferdinandy P., Puskás L.G. (2003). Effect of Classic Preconditioning on the Gene Expression Pattern of Rat Hearts: A DNA Microarray Study. FEBS Lett..

[45] Chen S., Zhang Y., Lighthouse J.K., Mickelsen D.M., Wu J., Yao P., Small E.M., Yan C. (2020). A Novel Role of Cyclic Nucleotide Phosphodiesterase 10A in Pathological Cardiac Remodeling and Dysfunction. Circulation.

[46] Hashimoto T., Kim G.E., Tunin R.S., Adesiyun T., Hsu S., Nakagawa R., Zhu G., O’Brien J.J., Hendrick J.P., Davis R.E. (2018). Acute Enhancement of Cardiac Function by Phosphodiesterase Type 1 Inhibition: Translational Study in the Dog and Rabbit. Circulation.

[47] Richter W., Xie M., Scheitrum C., Krall J., Movsesian M.A., Conti M. (2011). Conserved Expression and Functions of PDE4 in Rodent and Human Heart. Basic. Res. Cardiol..

[48] Leroy J., Richter W., Mika D., Castro L.R.V., Abi-Gerges A., Xie M., Scheitrum C., Lefebvre F., Schittl J., Mateo P. (2011). Phosphodiesterase 4B in the Cardiac L-Type Ca^2+^ Channel Complex Regulates Ca^2+^ Current and Protects against Ventricular Arrhythmias in Mice. J. Clin. Investig..

[49] Bhat A., Ray B., Mahalakshmi A.M., Tuladhar S., Nandakumar D., Srinivasan M., Essa M.M., Chidambaram S.B., Guillemin G.J., Sakharkar M.K. (2020). Phosphodiesterase-4 Enzyme as a Therapeutic Target in Neurological Disorders. Pharmacol. Res..

[50] Zhang K., Farooqui S.M., O’Donnell J.M. (1999). Ontogeny of Rolipram-Sensitive, Low-Km, Cyclic AMP-Specific Phosphodiesterase in Rat Brain. Dev. Brain Res..

[51] Rybalkin S.D., Hinds T.R., Beavo J.A., Krieg T., Lukowski R. (2013). Enzyme Assays for cGMP Hydrolyzing Phosphodiesterases. Guanylate Cyclase and Cyclic GMP.

[52] Xu R., Fu J., Hu Y., Yang X., Tao X., Chen L., Huang K., Fu Q. (2022). Roflumilast-Mediated Phosphodiesterase 4D Inhibition Reverses Diabetes-Associated Cardiac Dysfunction and Remodeling: Effects Beyond Glucose Lowering. Diabetes.

[53] Qasim H., Rajaei M., Xu Y., Reyes-Alcaraz A., Abdelnasser H.Y., Stewart M.D., Lahiri S.K., Wehrens X.H.T., McConnell B.K. (2024). AKAP12 Upregulation Associates with PDE8A to Accelerate Cardiac Dysfunction. Circ. Res..

[54] Grammatika Pavlidou N., Dobrev S., Beneke K., Reinhardt F., Pecha S., Jacquet E., Abu-Taha I.H., Schmidt C., Voigt N., Kamler M. (2023). Phosphodiesterase 8 Governs cAMP/PKA-Dependent Reduction of L-Type Calcium Current in Human Atrial Fibrillation: A Novel Arrhythmogenic Mechanism. Eur. Heart J..

[55] Hutchings D.C., Anderson S.G., Caldwell J.L., Trafford A.W. (2018). Phosphodiesterase-5 Inhibitors and the Heart: Compound Cardioprotection?. Heart.

[56] Gong W., Yan M., Chen J., Chaugai S., Chen C., Wang D. (2014). Chronic Inhibition of Cyclic Guanosine Monophosphate-Specific Phosphodiesterase 5 Prevented Cardiac Fibrosis through Inhibition of Transforming Growth Factor β-Induced Smad Signaling. Front. Med..

[57] Wang P., Li Z., Cai S., Li J., He P., Huang Y., Feng G., Luo H., Chen S., Liu P. (2017). C33(S), a Novel PDE9A Inhibitor, Protects against Rat Cardiac Hypertrophy through Upregulating cGMP Signaling. Acta Pharmacol. Sin..

[58] Lee D.I., Zhu G., Sasaki T., Cho G.-S., Hamdani N., Holewinski R., Jo S.-H., Danner T., Zhang M., Rainer P.P. (2015). Phosphodiesterase 9A Controls Nitric-Oxide-Independent cGMP and Hypertrophic Heart Disease. Nature.

[59] Vandeput F., Wolda S.L., Krall J., Hambleton R., Uher L., McCaw K.N., Radwanski P.B., Florio V., Movsesian M.A. (2007). Cyclic Nucleotide Phosphodiesterase PDE1C1 in Human Cardiac Myocytes. J. Biol. Chem..

[60] Miller C.L., Cai Y., Oikawa M., Thomas T., Dostmann W.R., Zaccolo M., Fujiwara K., Yan C. (2011). Cyclic Nucleotide Phosphodiesterase 1A: A Key Regulator of Cardiac Fibroblast Activation and Extracellular Matrix Remodeling in the Heart. Basic Res. Cardiol..

[61] Monterisi S., Lobo M.J., Livie C., Castle J.C., Weinberger M., Baillie G., Surdo N.C., Musheshe N., Stangherlin A., Gottlieb E. (2017). PDE2A2 Regulates Mitochondria Morphology and Apoptotic Cell Death via Local Modulation of cAMP/PKA Signalling. eLife.

[62] Sadek M.S., Cachorro E., El-Armouche A., Kämmerer S. (2020). Therapeutic Implications for PDE2 and cGMP/cAMP Mediated Crosstalk in Cardiovascular Diseases. Int. J. Mol. Sci..

[63] Movsesian M., Ahmad F., Hirsch E. (2018). Functions of PDE3 Isoforms in Cardiac Muscle. J. Cardiovasc. Dev. Dis..

[64] Beca S., Ahmad F., Shen W., Liu J., Makary S., Polidovitch N., Sun J., Hockman S., Chung Y.W., Movsesian M. (2013). Phosphodiesterase Type 3A Regulates Basal Myocardial Contractility Through Interacting with Sarcoplasmic Reticulum Calcium ATPase Type 2a Signaling Complexes in Mouse Heart. Circ. Res..

[65] Kamel R., Leroy J., Vandecasteele G., Fischmeister R. (2023). Cyclic Nucleotide Phosphodiesterases as Therapeutic Targets in Cardiac Hypertrophy and Heart Failure. Nat. Rev. Cardiol..

[66] Jäger R., Russwurm C., Schwede F., Genieser H.-G., Koesling D., Russwurm M. (2012). Activation of PDE10 and PDE11 Phosphodiesterases. J. Biol. Chem..

[67] Conti M., Richter W., Mehats C., Livera G., Park J.-Y., Jin C. (2003). Cyclic AMP-Specific PDE4 Phosphodiesterases as Critical Components of Cyclic AMP Signaling. J. Biol. Chem..

[68] Shi Q., Li M., Mika D., Fu Q., Kim S., Phan J., Shen A., Vandecasteele G., Xiang Y.K. (2017). Heterologous Desensitization of Cardiac β-Adrenergic Signal via Hormone-Induced βAR/Arrestin/PDE4 Complexes. Cardiovasc. Res..

[69] De Arcangelis V., Liu R., Soto D., Xiang Y. (2009). Differential Association of Phosphodiesterase 4D Isoforms with Β2-Adrenoceptor in Cardiac Myocytes. J. Biol. Chem..

[70] Richter W., Day P., Agrawal R., Bruss M.D., Granier S., Wang Y.L., Rasmussen S.G.F., Horner K., Wang P., Lei T. (2008). Signaling from Β1- and Β2-Adrenergic Receptors Is Defined by Differential Interactions with PDE4. EMBO J..

[71] Kajimoto K., Hagiwara N., Kasanuki H., Hosoda S. (1997). Contribution of Phosphodiesterase Isozymes to the Regulation of the L-type Calcium Current in Human Cardiac Myocytes. Br. J. Pharmacol..

[72] Molina C.E., Leroy J., Richter W., Xie M., Scheitrum C., Lee I.-O., Maack C., Rucker-Martin C., Donzeau-Gouge P., Verde I. (2012). Cyclic Adenosine Monophosphate Phosphodiesterase Type 4 Protects Against Atrial Arrhythmias. J. Am. Coll. Cardiol..

[73] Berisha F., Götz K.R., Wegener J.W., Brandenburg S., Subramanian H., Molina C.E., Rüffer A., Petersen J., Bernhardt A., Girdauskas E. (2021). cAMP Imaging at Ryanodine Receptors Reveals β_2_ -Adrenoceptor Driven Arrhythmias. Circ. Res..

[74] Sin Y.Y., Edwards H.V., Li X., Day J.P., Christian F., Dunlop A.J., Adams D.R., Zaccolo M., Houslay M.D., Baillie G.S. (2011). Disruption of the Cyclic AMP Phosphodiesterase-4 (PDE4)–HSP20 Complex Attenuates the β-Agonist Induced Hypertrophic Response in Cardiac Myocytes. J. Mol. Cell. Cardiol..

[75] Cedervall P., Aulabaugh A., Geoghegan K.F., McLellan T.J., Pandit J. (2015). Engineered Stabilization and Structural Analysis of the Autoinhibited Conformation of PDE4. Proc. Natl. Acad. Sci. USA.

[76] Richter W., Conti M. (2002). Dimerization of the Type 4 cAMP-Specific Phosphodiesterases Is Mediated by the Upstream Conserved Regions (UCRs). J. Biol. Chem..

[77] MacKenzie S.J., Baillie G.S., McPhee I., MacKenzie C., Seamons R., McSorley T., Millen J., Beard M.B., Van Heeke G., Houslay M.D. (2002). Long PDE4 cAMP Specific Phosphodiesterases Are Activated by Protein Kinase A-mediated Phosphorylation of a Single Serine Residue in Upstream Conserved Region 1 (UCR1). Br. J. Pharmacol..

[78] Sette C., Vicini E., Conti M. (1994). The ratPDE3/IVd Phosphodiesterase Gene Codes for Multiple Proteins Differentially Activated by cAMP-Dependent Protein Kinase. J. Biol. Chem..

[79] Sette C., Conti M. (1996). Phosphorylation and Activation of a cAMP-Specific Phosphodiesterase by the cAMP-Dependent Protein Kinase. J. Biol. Chem..

[80] Alvarez R., Sette C., Yang D., Eglen R.M., Wilhelm R., Shelton E.R., Conti M. (1995). Activation and Selective Inhibition of a Cyclic AMP-Specific Phosphodiesterase, PDE-4D3. Mol. Pharmacol..

[81] Hoffmann R., Wilkinson I.R., McCallum J.F., Engels P., Houslay M.D. (1998). cAMP-Specific Phosphodiesterase HSPDE4D3 Mutants Which Mimic Activation and Changes in Rolipram Inhibition Triggered by Protein Kinase A Phosphorylation of Ser-54: Generation of a Molecular Model. Biochem. J..

[82] Beard M.B., Olsen A.E., Jones R.E., Erdogan S., Houslay M.D., Bolger G.B. (2000). UCR1 and UCR2 Domains Unique to the cAMP-Specific Phosphodiesterase Family Form a Discrete Module via Electrostatic Interactions. J. Biol. Chem..

[83] MacKenzie S.J., Baillie G.S., McPhee I., Bolger G.B., Houslay M.D. (2000). ERK2 Mitogen-Activated Protein Kinase Binding, Phosphorylation, and Regulation of the PDE4D cAMP-Specific Phosphodiesterases. J. Biol. Chem..

[84] Lenhard J.M., Kassel D.B., Rocque W.J., Hamacher L., Holmes W.D., Patel I., Hoffman C., Luther M. (1996). Phosphorylation of a cAMP-Specific Phosphodiesterase (HSPDE4B2B) by Mitogen-Activated Protein Kinase. Biochem. J..

[85] Hoffmann R., Baillie G.S., MacKenzie S.J., Yarwood S.J., Houslay M.D. (1999). The MAP Kinase ERK2 Inhibits the Cyclic AMP-Specific Phosphodiesterase HSPDE4D3 by Phosphorylating It at Ser579. EMBO J..

[86] Baillie G.S., MacKenzie S.J., McPhee I., Houslay M.D. (2000). Sub-family Selective Actions in the Ability of Erk2 MAP Kinase to Phosphorylate and Regulate the Activity of PDE4 Cyclic AMP-specific Phosphodiesterases. Br. J. Pharmacol..

[87] Fox D., Burgin A.B., Gurney M.E. (2014). Structural Basis for the Design of Selective Phosphodiesterase 4B Inhibitors. Cell. Signal..

[88] Jumper J., Evans R., Pritzel A., Green T., Figurnov M., Ronneberger O., Tunyasuvunakool K., Bates R., Žídek A., Potapenko A. (2021). Highly Accurate Protein Structure Prediction with AlphaFold. Nature.

[89] Varadi M., Bertoni D., Magana P., Paramval U., Pidruchna I., Radhakrishnan M., Tsenkov M., Nair S., Mirdita M., Yeo J. (2024). AlphaFold Protein Structure Database in 2024: Providing Structure Coverage for over 214 Million Protein Sequences. Nucleic Acids Res..

[90] Schrödinger, LLC (2015). The PyMOL Molecular Graphics System, Version 1.8.

[91] Leroy J., Abi-Gerges A., Nikolaev V.O., Richter W., Lechêne P., Mazet J.-L., Conti M., Fischmeister R., Vandecasteele G. (2008). Spatiotemporal Dynamics of β-Adrenergic cAMP Signals and L-Type Ca^2+^ Channel Regulation in Adult Rat Ventricular Myocytes: Role of Phosphodiesterases. Circ. Res..

[92] Kerfant B.-G., Zhao D., Lorenzen-Schmidt I., Wilson L.S., Cai S., Chen S.R.W., Maurice D.H., Backx P.H. (2007). PI3Kγ Is Required for PDE4, Not PDE3, Activity in Subcellular Microdomains Containing the Sarcoplasmic Reticular Calcium ATPase in Cardiomyocytes. Circ. Res..

[93] Lehnart S.E., Wehrens X.H.T., Reiken S., Warrier S., Belevych A.E., Harvey R.D., Richter W., Jin S.-L.C., Conti M., Marks A.R. (2005). Phosphodiesterase 4D Deficiency in the Ryanodine-Receptor Complex Promotes Heart Failure and Arrhythmias. Cell.

[94] Johnson W.B., Katugampola S., Able S., Napier C., Harding S.E. (2012). Profiling of cAMP and cGMP Phosphodiesterases in Isolated Ventricular Cardiomyocytes from Human Hearts: Comparison with Rat and Guinea Pig. Life Sci..

[95] Reinhardt R.R., Chin E., Zhou J., Taira M., Murata T., Manganiello V.C., Bondy C.A. (1995). Distinctive Anatomical Patterns of Gene Expression for cGMP-Inhibited Cyclic Nucleotide Phosphodiesterases. J. Clin. Investig..

[96] Wechsler J., Choi Y.-H., Krall J., Ahmad F., Manganiello V.C., Movsesian M.A. (2002). Isoforms of Cyclic Nucleotide Phosphodiesterase PDE3A in Cardiac Myocytes. J. Biol. Chem..

[97] Reeves M.L., Leigh B.K., England P.J. (1987). The Identification of a New Cyclic Nucleotide Phosphodiesterase Activity in Human and Guinea-Pig Cardiac Ventricle. Implications for the Mechanism of Action of Selective Phosphodiesterase Inhibitors. Biochem. J..

[98] Muller B., Stoclet J.-C., Lugnier C. (1992). Cytosolic and Membrane-Bound Cyclic Nucleotide Phosphodiesterases from Guinea Pig Cardiac Ventricles. Eur. J. Pharmacol. Mol. Pharmacol..

[99] Patrucco E., Albergine M.S., Santana L.F., Beavo J.A. (2010). Phosphodiesterase 8A (PDE8A) Regulates Excitation–Contraction Coupling in Ventricular Myocytes. J. Mol. Cell. Cardiol..

[100] Subramanian H., Nikolaev V.O. (2024). AKAP12 Overexpression Affects Cardiac Function via PDE8. Circ. Res..

[101] Ismaili D., Petersen J., Schulz C., Eschenhagen T., Koivumäki J.T., Christ T. (2024). PDE8 Inhibition and Its Impact on I Ca,L in Persistent Atrial Fibrillation: Evaluation of PDE8 as a Potential Drug Target. J. Cardiovasc. Pharmacol..

[102] Willoughby D., Baillie G.S., Lynch M.J., Ciruela A., Houslay M.D., Cooper D.M.F. (2007). Dynamic Regulation, Desensitization, and Cross-Talk in Discrete Subcellular Microdomains during Β2-Adrenoceptor and Prostanoid Receptor cAMP Signaling. J. Biol. Chem..

[103] Barnes A.P., Livera G., Huang P., Sun C., O’Neal W.K., Conti M., Stutts M.J., Milgram S.L. (2005). Phosphodiesterase 4D Forms a cAMP Diffusion Barrier at the Apical Membrane of the Airway Epithelium. J. Biol. Chem..

[104] McCahill A., Campbell L., McSorley T., Sood A., Lynch M.J., Li X., Yan C., Baillie G.S., Houslay M.D. (2008). In Cardiac Myocytes, cAMP Elevation Triggers the down-Regulation of Transcripts and Promoter Activity for Cyclic AMP Phosphodiesterase-4A10 (PDE4A10). Cell. Signal..

[105] Mika D., Richter W., Westenbroek R.E., Catterall W.A., Conti M. (2014). PDE4B Mediates Local Feedback Regulation of Β1-Adrenergic cAMP Signaling in a Sarcolemmal Compartment of Cardiac Myocytes. J. Cell Sci..

[106] Willoughby D., Wong W., Schaack J., Scott J.D., Cooper D.M.F. (2006). An Anchored PKA and PDE4 Complex Regulates Subplasmalemmal cAMP Dynamics. EMBO J..

[107] Houslay M.D., Baillie G.S., Maurice D.H. (2007). cAMP-Specific Phosphodiesterase-4 Enzymes in the Cardiovascular System: A Molecular Toolbox for Generating Compartmentalized cAMP Signaling. Circ. Res..

[108] Lynch M.J., Baillie G.S., Houslay M.D. (2007). cAMP-Specific Phosphodiesterase-4D5 (PDE4D5) Provides a Paradigm for Understanding the Unique Non-Redundant Roles That PDE4 Isoforms Play in Shaping Compartmentalized cAMP Cell Signalling. Biochem. Soc. Trans..

[109] Kyurkchieva E., Baillie G.S. (2023). Short PDE4 Isoforms as Drug Targets in Disease. Front. Biosci. (Landmark Ed).

[110] Karam S., Margaria J.P., Bourcier A., Mika D., Varin A., Bedioune I., Lindner M., Bouadjel K., Dessillons M., Gaudin F. (2020). Cardiac Overexpression of PDE4B Blunts β-Adrenergic Response and Maladaptive Remodeling in Heart Failure. Circulation.

[111] Mika D., Bobin P., Pomérance M., Lechêne P., Westenbroek R.E., Catterall W.A., Vandecasteele G., Leroy J., Fischmeister R. (2013). Differential Regulation of Cardiac Excitation–Contraction Coupling by cAMP Phosphodiesterase Subtypes. Cardiovasc. Res..

[112] Molenaar P., Christ T., Hussain R.I., Engel A., Berk E., Gillette K.T., Chen L., Galindo-Tovar A., Krobert K.A., Ravens U. (2013). PDE3, but Not PDE4, Reduces β_1_ - and β_2_ -adrenoceptor-mediated Inotropic and Lusitropic Effects in Failing Ventricle from Metoprolol-treated Patients. Br. J. Pharmacol..

[113] Bobin P., Varin A., Lefebvre F., Fischmeister R., Vandecasteele G., Leroy J. (2016). Calmodulin Kinase II Inhibition Limits the Pro-Arrhythmic Ca^2+^ Waves Induced by cAMP-Phosphodiesterase Inhibitors. Cardiovasc. Res..

[114] Wright P.T., Bhogal N.K., Diakonov I., Pannell L.M.K., Perera R.K., Bork N.I., Schobesberger S., Lucarelli C., Faggian G., Alvarez-Laviada A. (2018). Cardiomyocyte Membrane Structure and cAMP Compartmentation Produce Anatomical Variation in β2AR-cAMP Responsiveness in Murine Hearts. Cell Rep..

[115] Molina C.E., Johnson D.M., Mehel H., Spätjens R.L.H.M.G., Mika D., Algalarrondo V., Slimane Z.H., Lechêne P., Abi-Gerges N., Van Der Linde H.J. (2014). Interventricular Differences in β-Adrenergic Responses in the Canine Heart: Role of Phosphodiesterases. JAHA.

[116] La Gerche A., Heidbüchel H., Burns A.T., Mooney D.J., Taylor A.J., Pfluger H.B., Inder W.J., Macisaac A.I., Prior D.L. (2011). Disproportionate Exercise Load and Remodeling of the Athlete’s Right Ventricle. Med. Sci. Sports Exerc..

[117] Glukhov A.V., Balycheva M., Sanchez-Alonso J.L., Ilkan Z., Alvarez-Laviada A., Bhogal N., Diakonov I., Schobesberger S., Sikkel M.B., Bhargava A. (2015). Direct Evidence for Microdomain-Specific Localization and Remodeling of Functional L-Type Calcium Channels in Rat and Human Atrial Myocytes. Circulation.

[118] Judina A., Niglas M., Leonov V., Kirkby N.S., Diakonov I., Wright P.T., Zhao L., Mitchell J.A., Gorelik J. (2023). Pulmonary Hypertension-Associated Right Ventricular Cardiomyocyte Remodelling Reduces Treprostinil Function. Cells.

[119] Fu J., Mansfield C., Diakonov I., Judina A., Delahaye M., Bhogal N., Sanchez-Alonso J.L., Kamp T., Gorelik J. (2025). Stretch Regulation of Β2-Adrenoceptor Signalling in Cardiomyocytes Requires Caveolae. Cardiovasc. Res..

[120] Wright P.T., Nikolaev V.O., O’Hara T., Diakonov I., Bhargava A., Tokar S., Schobesberger S., Shevchuk A.I., Sikkel M.B., Wilkinson R. (2014). Caveolin-3 Regulates Compartmentation of Cardiomyocyte Beta2-Adrenergic Receptor-Mediated cAMP Signaling. J. Mol. Cell. Cardiol..

[121] Bedioune I., Lefebvre F., Lechêne P., Varin A., Domergue V., Kapiloff M.S., Fischmeister R., Vandecasteele G. (2018). PDE4 and mAKAPβ Are Nodal Organizers of Β2-ARs Nuclear PKA Signalling in Cardiac Myocytes. Cardiovasc. Res..

[122] Xiang Y., Naro F., Zoudilova M., Jin S.-L.C., Conti M., Kobilka B. (2005). Phosphodiesterase 4D Is Required for β_2_ Adrenoceptor Subtype-Specific Signaling in Cardiac Myocytes. Proc. Natl. Acad. Sci. USA.

[123] Epstein P.M., Andrenyak D.M., Smith C.J., Pappano A.J. (1987). Ontogenetic Changes in Adenylate Cyclase, Cyclic AMP Phosphodiesterase and Calmodulin in Chick Ventricular Myocardium. Biochem. J..

[124] Higgins D., Pappano A.J. (1981). Developmental Changes in the Sensitivity of the Chick Embryo Ventricle to Beta-Adrenergic Agonist during Adrenergic Innervation. Circ. Res..

[125] Berthouze-Duquesnes M., Lucas A., Saulière A., Sin Y.Y., Laurent A.-C., Galés C., Baillie G., Lezoualc’h F. (2013). Specific Interactions between Epac1, β-Arrestin2 and PDE4D5 Regulate β-Adrenergic Receptor Subtype Differential Effects on Cardiac Hypertrophic Signaling. Cell. Signal..

[126] Kraft A.E., Bork N.I., Subramanian H., Pavlaki N., Failla A.V., Zobiak B., Conti M., Nikolaev V.O. (2024). Phosphodiesterases 4B and 4D Differentially Regulate cAMP Signaling in Calcium Handling Microdomains of Mouse Hearts. Cells.

[127] Wright P.T., Sanchez-Alonso J.L., Lucarelli C., Alvarez-Laviada A., Poulet C.E., Bello S.O., Faggian G., Terracciano C.M., Gorelik J. (2018). Partial Mechanical Unloading of the Heart Disrupts L-Type Calcium Channel and Beta-Adrenoceptor Signaling Microdomains. Front. Physiol..

[128] Rochais F., Abi-Gerges A., Horner K., Lefebvre F., Cooper D.M.F., Conti M., Fischmeister R., Vandecasteele G. (2006). A Specific Pattern of Phosphodiesterases Controls the cAMP Signals Generated by Different G_s_-Coupled Receptors in Adult Rat Ventricular Myocytes. Circ. Res..

[129] Verde I., Vandecasteele G., Lezoualc’h F., Fischmeister R. (1999). Characterization of the Cyclic Nucleotide Phosphodiesterase Subtypes Involved in the Regulation of the L-type Ca^2+^ Current in Rat Ventricular Myocytes. Br. J. Pharmacol..

[130] Beca S., Helli P.B., Simpson J.A., Zhao D., Farman G.P., Jones P.P., Tian X., Wilson L.S., Ahmad F., Chen S.R.W. (2011). Phosphodiesterase 4D Regulates Baseline Sarcoplasmic Reticulum Ca^2+^ Release and Cardiac Contractility, Independently of L-Type Ca^2+^ Current. Circ. Res..

[131] Schwinger R.H.G., Münch G., Bölck B., Karczewski P., Krause E.-G., Erdmann E. (1999). Reduced Ca^2+^-Sensitivity of SERCA 2a in Failing Human Myocardium Due to Reduced Serin-16 Phospholamban Phoshorylation. J. Mol. Cell. Cardiol..

[132] Sande J.B., Sjaastad I., Hoen I.B., Bøkenes J., Tønnessen T., Holt E., Lunde P.K., Christensen G. (2002). Reduced Level of Serine16 Phosphorylated Phospholamban in the Failing Rat Myocardium: A Major Contributor to Reduced SERCA2 Activity. Cardiovasc. Res..

[133] Minamisawa S., Hoshijima M., Chu G., Ward C.A., Frank K., Gu Y., Martone M.E., Wang Y., Ross J., Kranias E.G. (1999). Chronic Phospholamban–Sarcoplasmic Reticulum Calcium ATPase Interaction Is the Critical Calcium Cycling Defect in Dilated Cardiomyopathy. Cell.

[134] Sprenger J.U., Perera R.K., Steinbrecher J.H., Lehnart S.E., Maier L.S., Hasenfuss G., Nikolaev V.O. (2015). In Vivo Model with Targeted cAMP Biosensor Reveals Changes in Receptor–Microdomain Communication in Cardiac Disease. Nat. Commun..

[135] Lugnier C., Keravis T., Le Bec A., Pauvert O., Proteau S., Rousseau E. (1999). Characterization of Cyclic Nucleotide Phosphodiesterase Isoforms Associated to Isolated Cardiac Nuclei. Biochim. Biophys. Acta (BBA) Gen. Subj..

[136] Haj Slimane Z., Bedioune I., Lechêne P., Varin A., Lefebvre F., Mateo P., Domergue-Dupont V., Dewenter M., Richter W., Conti M. (2014). Control of Cytoplasmic and Nuclear Protein Kinase A by Phosphodiesterases and Phosphatases in Cardiac Myocytes. Cardiovasc. Res..

[137] Wang L., Burmeister B.T., Johnson K.R., Baillie G.S., Karginov A.V., Skidgel R.A., O’Bryan J.P., Carnegie G.K. (2015). UCR1C Is a Novel Activator of Phosphodiesterase 4 (PDE4) Long Isoforms and Attenuates Cardiomyocyte Hypertrophy. Cell. Signal..

[138] McCartney S., Little B.M., Langeberg L.K., Scott J.D. (1995). Cloning and Characterization of A-Kinase Anchor Protein 100 (AKAP100). J. Biol. Chem..

[139] Huang L.J., Durick K., Weiner J.A., Chun J., Taylor S.S. (1997). Identification of a Novel Protein Kinase A Anchoring Protein That Binds Both Type I and Type II Regulatory Subunits. J. Biol. Chem..

[140] Huang L.J., Durick K., Weiner J.A., Chun J., Taylor S.S. (1997). D-AKAP2, a Novel Protein Kinase A Anchoring Protein with a Putative RGS Domain. Proc. Natl. Acad. Sci. USA.

[141] Ginsberg M.D., Feliciello A., Jones J.K., Avvedimento E.V., Gottesman M.E. (2003). PKA-Dependent Binding of mRNA to the Mitochondrial AKAP121 Protein. J. Mol. Biol..

[142] Trendelenburg G., Hummel M., Riecken E.-O., Hanski C. (1996). Molecular Characterization of AKAP149, a Novel A Kinase Anchor Protein with a KH Domain. Biochem. Biophys. Res. Commun..

[143] Lin R.-Y., Moss S.B., Rubin C.S. (1995). Characterization of S-AKAP84, a Novel Developmentally Regulated A Kinase Anchor Protein of Male Germ Cells. J. Biol. Chem..

[144] Schiattarella G.G., Boccella N., Paolillo R., Cattaneo F., Trimarco V., Franzone A., D’Apice S., Giugliano G., Rinaldi L., Borzacchiello D. (2018). Loss of Akap1 Exacerbates Pressure Overload-Induced Cardiac Hypertrophy and Heart Failure. Front. Physiol..

[145] Xiang H., Xu H., Tan B., Yi Q., Zhang X., Wang R., Chen T., Xie Q., Tian J., Zhu J. (2023). AKAP1 Regulates Mitochondrial Dynamics during the Fatty-Acid-Promoted Maturation of Human-Induced Pluripotent Stem Cell-Derived Cardiomyocytes as Indicated by Proteomics Sequencing. Int. J. Mol. Sci..

[146] Maric D., Paterek A., Delaunay M., López I.P., Arambasic M., Diviani D. (2021). A-Kinase Anchoring Protein 2 Promotes Protection against Myocardial Infarction. Cells.

[147] Kashishian A., Howard M., Loh C., Gallatin W.M., Hoekstra M.F., Lai Y. (1998). AKAP79 Inhibits Calcineurin through a Site Distinct from the Immunophilin-Binding Region. J. Biol. Chem..

[148] Hoshi N., Zhang J.-S., Omaki M., Takeuchi T., Yokoyama S., Wanaverbecq N., Langeberg L.K., Yoneda Y., Scott J.D., Brown D.A. (2003). AKAP150 Signaling Complex Promotes Suppression of the M-Current by Muscarinic Agonists. Nat. Neurosci..

[149] Li Y., Ndubuka C., Rubin C.S. (1996). A Kinase Anchor Protein 75 Targets Regulatory (RII) Subunits of cAMP-Dependent Protein Kinase II to the Cortical Actin Cytoskeleton in Non-Neuronal Cells. J. Biol. Chem..

[150] Nichols C.B., Rossow C.F., Navedo M.F., Westenbroek R.E., Catterall W.A., Santana L.F., McKnight G.S. (2010). Sympathetic Stimulation of Adult Cardiomyocytes Requires Association of AKAP5 With a Subpopulation of L-Type Calcium Channels. Circ. Res..

[151] Surdo N.C., Berrera M., Koschinski A., Brescia M., Machado M.R., Carr C., Wright P., Gorelik J., Morotti S., Grandi E. (2017). FRET Biosensor Uncovers cAMP Nano-Domains at β-Adrenergic Targets That Dictate Precise Tuning of Cardiac Contractility. Nat. Commun..

[152] Li J., Negro A., Lopez J., Bauman A.L., Henson E., Dodge-Kafka K., Kapiloff M.S. (2010). The mAKAPβ Scaffold Regulates Cardiac Myocyte Hypertrophy via Recruitment of Activated Calcineurin. J. Mol. Cell. Cardiol..

[153] Kapiloff M.S., Schillace R.V., Westphal A.M., Scott J.D. (1999). mAKAP: An A-Kinase Anchoring Protein Targeted to the Nuclear Membrane of Differentiated Myocytes. J. Cell Sci..

[154] Hakem Zadeh F., Teng A.C.T., Kuzmanov U., Chambers P.J., Tupling A.R., Gramolini A.O. (2019). AKAP6 and Phospholamban Colocalize and Interact in HEK-293T Cells and Primary Murine Cardiomyocytes. Physiol. Rep..

[155] Vergarajauregui S., Becker R., Steffen U., Sharkova M., Esser T., Petzold J., Billing F., Kapiloff M.S., Schett G., Thievessen I. (2020). AKAP6 Orchestrates the Nuclear Envelope Microtubule-Organizing Center by Linking Golgi and Nucleus via AKAP9. eLife.

[156] Gray P.C., Johnson B.D., Westenbroek R.E., Hays L.G., Yates J.R., Scheuer T., Catterall W.A., Murphy B.J. (1998). Primary Structure and Function of an A Kinase Anchoring Protein Associated with Calcium Channels. Neuron.

[157] Gold M.G., Smith F.D., Scott J.D., Barford D. (2008). AKAP18 Contains a Phosphoesterase Domain That Binds AMP. J. Mol. Biol..

[158] Lygren B., Carlson C.R., Santamaria K., Lissandron V., McSorley T., Litzenberg J., Lorenz D., Wiesner B., Rosenthal W., Zaccolo M. (2007). AKAP Complex Regulates Ca^2+^ Re-uptake into Heart Sarcoplasmic Reticulum. EMBO Rep..

[159] Singh A., Redden J.M., Kapiloff M.S., Dodge-Kafka K.L. (2011). The Large Isoforms of A-Kinase Anchoring Protein 18 Mediate the Phosphorylation of Inhibitor-1 by Protein Kinase A and the Inhibition of Protein Phosphatase 1 Activity. Mol. Pharmacol..

[160] Seyler C., Scherer D., Köpple C., Kulzer M., Korkmaz S., Xynogalos P., Thomas D., Kaya Z., Scholz E., Backs J. (2017). Role of Plasma Membrane-Associated AKAPs for the Regulation of Cardiac IK1 Current by Protein Kinase A. Naunyn-Schmiedeberg’s Arch. Pharmacol..

[161] Lin J.W., Wyszynski M., Madhavan R., Sealock R., Kim J.U., Sheng M. (1998). Yotiao, a Novel Protein of Neuromuscular Junction and Brain That Interacts with Specific Splice Variants of NMDA Receptor Subunit NR1. J. Neurosci..

[162] Shanks R.A., Steadman B.T., Schmidt P.H., Goldenring J.R. (2002). AKAP350 at the Golgi Apparatus. J. Biol. Chem..

[163] Gillingham A.K., Munro S. (2000). The PACT Domain, a Conserved Centrosomal Targeting Motif in the Coiled-coil Proteins AKAP450 and Pericentrin. EMBO Rep..

[164] Marx S.O., Kurokawa J., Reiken S., Motoike H., D’Armiento J., Marks A.R., Kass R.S. (2002). Requirement of a Macromolecular Signaling Complex for β Adrenergic Receptor Modulation of the KCNQ1-KCNE1 Potassium Channel. Science.

[165] Li Y., Chen L., Kass R.S., Dessauer C.W. (2012). The A-Kinase Anchoring Protein Yotiao Facilitates Complex Formation between Adenylyl Cyclase Type 9 and the IKs Potassium Channel in Heart. J. Biol. Chem..

[166] Nicolas C.S., Park K.-H., El Harchi A., Camonis J., Kass R.S., Escande D., Mérot J., Loussouarn G., Le Bouffant F., Baró I. (2008). I Ks Response to Protein Kinase A-Dependent KCNQ1 Phosphorylation Requires Direct Interaction with Microtubules. Cardiovasc. Res..

[167] Nauert J.B., Klauck T.M., Langeberg L.K., Scott J.D. (1997). Gravin, an Autoantigen Recognized by Serum from Myasthenia Gravis Patients, Is a Kinase Scaffold Protein. Curr. Biol..

[168] Xia W., Unger P., Miller L., Nelson J., Gelman I.H. (2001). The Src-Suppressed C Kinase Substrate, SSeCKS, Is a Potential Metastasis Inhibitor in Prostate Cancer. Cancer Res..

[169] Tao J. (2003). Protein Kinase A Regulates AKAP250 (Gravin) Scaffold Binding to the 2-Adrenergic Receptor. EMBO J..

[170] Li Y., Yu Q.-H., Chu Y., Wu W.-M., Song J.-X., Zhu X.-B., Wang Q. (2018). Blockage of AKAP12 Accelerates Angiotensin II (Ang II)-Induced Cardiac Injury in Mice by Regulating the Transforming Growth Factor Β1 (TGF-Β1) Pathway. Biochem. Biophys. Res. Commun..

[171] Diviani D., Soderling J., Scott J.D. (2001). AKAP-Lbc Anchors Protein Kinase A and Nucleates Gα12-Selective Rho-Mediated Stress Fiber Formation. J. Biol. Chem..

[172] Carnegie G.K., Soughayer J., Smith F.D., Pedroja B.S., Zhang F., Diviani D., Bristow M.R., Kunkel M.T., Newton A.C., Langeberg L.K. (2008). AKAP-Lbc Mobilizes a Cardiac Hypertrophy Signaling Pathway. Mol. Cell.

[173] Appert-Collin A., Cotecchia S., Nenniger-Tosato M., Pedrazzini T., Diviani D. (2007). The A-Kinase Anchoring Protein (AKAP)-Lbc-Signaling Complex Mediates A1 Adrenergic Receptor-Induced Cardiomyocyte Hypertrophy. Proc. Natl. Acad. Sci. USA.

[174] Caso S., Maric D., Arambasic M., Cotecchia S., Diviani D. (2017). AKAP-Lbc Mediates Protection against Doxorubicin-Induced Cardiomyocyte Toxicity. Biochim. Biophys. Acta (BBA) Mol. Cell Res..

[175] Perino A., Ghigo A., Ferrero E., Morello F., Santulli G., Baillie G.S., Damilano F., Dunlop A.J., Pawson C., Walser R. (2011). Integrating Cardiac PIP3 and cAMP Signaling through a PKA Anchoring Function of P110γ. Mol. Cell.

[176] Ghigo A., Murabito A., Sala V., Pisano A.R., Bertolini S., Gianotti A., Caci E., Montresor A., Premchandar A., Pirozzi F. (2022). A PI3Kγ Mimetic Peptide Triggers CFTR Gating, Bronchodilation, and Reduced Inflammation in Obstructive Airway Diseases. Sci. Transl. Med..

[177] Ghigo A., Perino A., Mehel H., Zahradníková A., Morello F., Leroy J., Nikolaev V.O., Damilano F., Cimino J., De Luca E. (2012). Phosphoinositide 3-Kinase γ Protects Against Catecholamine-Induced Ventricular Arrhythmia Through Protein Kinase A–Mediated Regulation of Distinct Phosphodiesterases. Circulation.

[178] Ndongson-Dongmo B., Heller R., Hoyer D., Brodhun M., Bauer M., Winning J., Hirsch E., Wetzker R., Schlattmann P., Bauer R. (2015). Phosphoinositide 3-Kinase Gamma Controls Inflammation-Induced Myocardial Depression via Sequential cAMP and iNOS Signalling. Cardiovasc. Res..

[179] Kang M., Otani Y., Guo Y., Yan J., Goult B.T., Howe A.K. (2024). The Focal Adhesion Protein Talin Is a Mechanically Gated A-Kinase Anchoring Protein. Proc. Natl. Acad. Sci. USA.

[180] Hirsch E., Katanaev V.L., Garlanda C., Azzolino O., Pirola L., Silengo L., Sozzani S., Mantovani A., Altruda F., Wymann M.P. (2000). Central Role for G Protein-Coupled Phosphoinositide 3-Kinase γ in Inflammation. Science.

[181] Li M., Sala V., De Santis M.C., Cimino J., Cappello P., Pianca N., Di Bona A., Margaria J.P., Martini M., Lazzarini E. (2018). Phosphoinositide 3-Kinase Gamma Inhibition Protects from Anthracycline Cardiotoxicity and Reduces Tumor Growth. Circulation.

[182] Elosegui-Artola A., Oria R., Chen Y., Kosmalska A., Pérez-González C., Castro N., Zhu C., Trepat X., Roca-Cusachs P. (2016). Mechanical Regulation of a Molecular Clutch Defines Force Transmission and Transduction in Response to Matrix Rigidity. Nat. Cell Biol..

[183] Haining A.W.M., Rahikainen R., Cortes E., Lachowski D., Rice A., Von Essen M., Hytönen V.P., Del Río Hernández A. (2018). Mechanotransduction in Talin through the Interaction of the R8 Domain with DLC1. PLoS Biol..

[184] Terrenoire C., Houslay M.D., Baillie G.S., Kass R.S. (2009). The Cardiac IKs Potassium Channel Macromolecular Complex Includes the Phosphodiesterase PDE4D3. J. Biol. Chem..

[185] Terrin A., Monterisi S., Stangherlin A., Zoccarato A., Koschinski A., Surdo N.C., Mongillo M., Sawa A., Jordanides N.E., Mountford J.C. (2012). PKA and PDE4D3 Anchoring to AKAP9 Provides Distinct Regulation of cAMP Signals at the Centrosome. J. Cell Biol..

[186] Kapiloff M.S., Jackson N., Airhart N. (2001). mAKAP and the Ryanodine Receptor Are Part of a Multi-Component Signaling Complex on the Cardiomyocyte Nuclear Envelope. J. Cell Sci..

[187] Carlisle Michel J.J., Dodge K.L., Wong W., Mayer N.C., Langeberg L.K., Scott J.D. (2004). PKA-Phosphorylation of PDE4D3 Facilitates Recruitment of the mAKAP Signalling Complex. Biochem. J..

[188] Dodge K.L. (2001). mAKAP Assembles a Protein Kinase A/PDE4 Phosphodiesterase cAMP Signaling Module. EMBO J..

[189] Dodge-Kafka K.L., Soughayer J., Pare G.C., Carlisle Michel J.J., Langeberg L.K., Kapiloff M.S., Scott J.D. (2005). The Protein Kinase A Anchoring Protein mAKAP Coordinates Two Integrated cAMP Effector Pathways. Nature.

[190] Fields L.A., Koschinski A., Zaccolo M. (2016). Sustained Exposure to Catecholamines Affects cAMP/PKA Compartmentalised Signalling in Adult Rat Ventricular Myocytes. Cell. Signal..

[191] Abi-Gerges A., Richter W., Lefebvre F., Mateo P., Varin A., Heymes C., Samuel J.-L., Lugnier C., Conti M., Fischmeister R. (2009). Decreased Expression and Activity of cAMP Phosphodiesterases in Cardiac Hypertrophy and Its Impact on β-Adrenergic cAMP Signals. Circ. Res..

[192] Qvigstad E., Moltzau L.R., Aronsen J.M., Nguyen C.H.T., Hougen K., Sjaastad I., Levy F.O., Skomedal T., Osnes J.-B. (2010). Natriuretic Peptides Increase Β1-Adrenoceptor Signalling in Failing Hearts through Phosphodiesterase 3 Inhibition. Cardiovasc. Res..

[193] Colombe A.-S., Pidoux G. (2021). Cardiac cAMP-PKA Signaling Compartmentalization in Myocardial Infarction. Cells.

[194] Pavlaki N., Froese A., Li W., De Jong K.A., Geertz B., Subramanian H., Mohagaonkar S., Luo X., Schubert M., Wiegmann R. (2024). Gene Therapy with Phosphodiesterases 2A and 4B Ameliorates Heart Failure and Arrhythmias by Improving Subcellular cAMP Compartmentation. Cardiovasc. Res..

[195] Abrenica B., AlShaaban M., Czubryt M.P. (2009). The A-Kinase Anchor Protein AKAP121 Is a Negative Regulator of Cardiomyocyte Hypertrophy. J. Mol. Cell. Cardiol..

[196] Zhang L., Malik S., Kelley G.G., Kapiloff M.S., Smrcka A.V. (2011). Phospholipase C∈ Scaffolds to Muscle-Specific A Kinase Anchoring Protein (mAKAPβ) and Integrates Multiple Hypertrophic Stimuli in Cardiac Myocytes. J. Biol. Chem..

[197] Dodge-Kafka K., Gildart M., Tokarski K., Kapiloff M.S. (2019). mAKAPβ Signalosomes—A Nodal Regulator of Gene Transcription Associated with Pathological Cardiac Remodeling. Cell. Signal..

[198] Li J., Aponte Paris S., Thakur H., Kapiloff M.S., Dodge-Kafka K.L. (2019). Muscle A-Kinase–Anchoring Protein-β–Bound Calcineurin Toggles Active and Repressive Transcriptional Complexes of Myocyte Enhancer Factor 2D. J. Biol. Chem..

[199] Kritzer M.D., Li J., Passariello C.L., Gayanilo M., Thakur H., Dayan J., Dodge-Kafka K., Kapiloff M.S. (2014). The Scaffold Protein Muscle A-Kinase Anchoring Protein β Orchestrates Cardiac Myocyte Hypertrophic Signaling Required for the Development of Heart Failure. Circ. Heart Fail..

[200] Lai P., Hille S.S., Subramanian H., Wiegmann R., Roser P., Müller O.J., Nikolaev V.O., De Jong K.A. (2024). Remodelling of cAMP Dynamics within the SERCA2a Microdomain in Heart Failure with Preserved Ejection Fraction Caused by Obesity and Type 2 Diabetes. Cardiovasc. Res..

[201] Soilness J.E., Maslen C., Webber S., Foster M., Raeburn D., Palfreyman M.N., Ashton M.J., Karlsson J. (1995). Suppression of Eosinophil Function by RP 73401, a Potent and Selective Inhibitor of Cyclic AMP-specific Phosphodiesterase: Comparison with Rolipram. Br. J Pharmacol..

[202] Rolan P., Hutchinson M., Johnson K. (2009). Ibudilast: A Review of Its Pharmacology, Efficacy and Safety in Respiratory and Neurological Disease. Expert. Opin. Pharmacother..

[203] Titus D.J., Wilson N.M., Alcazar O., Calixte D.A., Dietrich W.D., Gurney M.E., Atkins C.M. (2018). A Negative Allosteric Modulator of PDE4D Enhances Learning after Traumatic Brain Injury. Neurobiol. Learn. Mem..

[204] Gurney M.E., Nugent R.A., Mo X., Sindac J.A., Hagen T.J., Fox D., O’Donnell J.M., Zhang C., Xu Y., Zhang H.-T. (2019). Design and Synthesis of Selective Phosphodiesterase 4D (PDE4D) Allosteric Inhibitors for the Treatment of Fragile X Syndrome and Other Brain Disorders. J. Med. Chem..

[205] Zhang C., Xu Y., Chowdhary A., Fox D., Gurney M.E., Zhang H.-T., Auerbach B.D., Salvi R.J., Yang M., Li G. (2018). Memory Enhancing Effects of BPN14770, an Allosteric Inhibitor of Phosphodiesterase-4D, in Wild-Type and Humanized Mice. Neuropsychopharmacol..

[206] Burgin A.B., Magnusson O.T., Singh J., Witte P., Staker B.L., Bjornsson J.M., Thorsteinsdottir M., Hrafnsdottir S., Hagen T., Kiselyov A.S. (2010). Design of Phosphodiesterase 4D (PDE4D) Allosteric Modulators for Enhancing Cognition with Improved Safety. Nat. Biotechnol..

[207] McDowell L., Olin B. (2019). Crisaborole: A Novel Nonsteroidal Topical Treatment for Atopic Dermatitis. J. Pharm. Technol..

[208] Rabe K.F. (2011). Update on Roflumilast, a Phosphodiesterase 4 Inhibitor for the Treatment of Chronic Obstructive Pulmonary Disease. Br. J. Pharmacol..

[209] Schafer P.H., Parton A., Capone L., Cedzik D., Brady H., Evans J.F., Man H.-W., Muller G.W., Stirling D.I., Chopra R. (2014). Apremilast Is a Selective PDE4 Inhibitor with Regulatory Effects on Innate Immunity. Cell. Signal..

[210] Tralau-Stewart C.J., Williamson R.A., Nials A.T., Gascoigne M., Dawson J., Hart G.J., Angell A.D.R., Solanke Y.E., Lucas F.S., Wiseman J. (2011). GSK256066, an Exceptionally High-Affinity and Selective Inhibitor of Phosphodiesterase 4 Suitable for Administration by Inhalation: In Vitro, Kinetic, and In Vivo Characterization. J. Pharmacol. Exp. Ther..

[211] Warren R.B., Strober B., Silverberg J.I., Guttman E., Andres P., Felding J., Tutkunkardas D., Kjøller K., Sommer M.O.A., French L.E. (2023). Oral Orismilast: Efficacy and Safety in Moderate-to-severe Psoriasis and Development of Modified Release Tablets. Acad. Dermatol. Venereol..

[212] White W.B., Cooke G.E., Kowey P.R., Calverley P.M.A., Bredenbröker D., Goehring U.-M., Zhu H., Lakkis H., Mosberg H., Rowe P. (2013). Cardiovascular Safety in Patients Receiving Roflumilast for the Treatment of COPD. Chest.

[213] Dong C., Virtucio C., Zemska O., Baltazar G., Zhou Y., Baia D., Jones-Iatauro S., Sexton H., Martin S., Dee J. (2016). Treatment of Skin Inflammation with Benzoxaborole Phosphodiesterase Inhibitors: Selectivity, Cellular Activity, and Effect on Cytokines Associated with Skin Inflammation and Skin Architecture Changes. J. Pharmacol. Exp. Ther..

[214] Sin Y.Y., Giblin A., Judina A., Rujirachaivej P., Corral L.G., Glennon E., Tai Z.X., Feng T., Torres E., Zorn A. (2024). Targeted Protein Degradation of PDE4 Shortforms by a Novel Proteolysis Targeting Chimera. FEBS J..

[215] Murabito A., Cnudde S., Hirsch E., Ghigo A. (2020). Potential Therapeutic Applications of AKAP Disrupting Peptides. Clin. Sci..

[216] Della Sala A., Tasca L., Butnarasu C., Sala V., Prono G., Murabito A., Garbero O.V., Millo E., Terranova L., Blasi F. (2024). A Nonnatural Peptide Targeting the A-Kinase Anchoring Function of PI3Kγ for Therapeutic cAMP Modulation in Pulmonary Cells. J. Biol. Chem..

